# Supplementary material for: lncRNA 5430416N02Rik Promotes the Proliferation of Mouse Embryonic Stem Cells by Activating Mid1 Expression through 3D Chromatin Architecture
Source: Stem Cell Reports. 2020 Mar 10;14(3):493–505. doi: 10.1016/j.stemcr.2020.02.002 (PMC7066321; doi:10.1016/j.stemcr.2020.02.002)
Supplement: Document S1. Figures S1–S5 [file mmc1.pdf]

Stem Cell Reports, Volume *14*

Supplemental Information

lncRNA *5430416N02Rik* Promotes the Proliferation of Mouse Embryonic Stem Cells by Activating *Mid1* Expression through 3D Chromatin Architecture

Tong Zhao, Mingyang Cai, Man Liu, Guangsong Su, Daniel An, Byoungsan Moon, Guochang Lyu, Yibo Si, Lingyi Chen, and Wange Lu

Supplemental Information

Supplemental Figures and Legends

Figure S1. Analysis of RIP-seq profiles. (Related to Figure 1)

Figure S2. The *5430416N02Rik* promoter is a chromatin interaction hub. (Related to Figure 2)

Figure S3. Both homozygous ΔPG and ΔG ESCs show reduced growth rates. (Related to Figure 3)

Figure S4. The transcriptional profile is perturbed more in homozygous ΔPG ESCs than in homozygous ΔG ESCs. (Related to Figure 3)

Figure S5. Both *5430416N02Rik* DNA and RNA interact with the *Mid1* locus. (Related to Figure 4)

Supplemental Tables

Table S1. Klf4-bound RNAs identified by RIP-seq; Lists of Klf4-bound lncRNAs, lncRNAs regulating ESC transcriptome, and lncRNAs activated during reprogramming. (Table S1.xlsx)

Table S2 *5430416N02Rik*-interacting sites and regions identified by 4C-seq; *5430416N02Rik*-interacting sites in heterozygous and homozygous ΔG ESCs identified by Capture-C seq; Differential *5430416N02Rik*-interacting sites between heterozygous and homozygous ΔG ESCs identified by Capture-C seq. (Table S2.xlsx)

Table S3. Differentially expressed genes in heterozygous and homozygous ΔPG and ΔG ESCs, as well as RA treated ESCs identified by RNA-seq; List of primers used in the study. (Table S3.xlsx)

Supplemental Figures and Legends

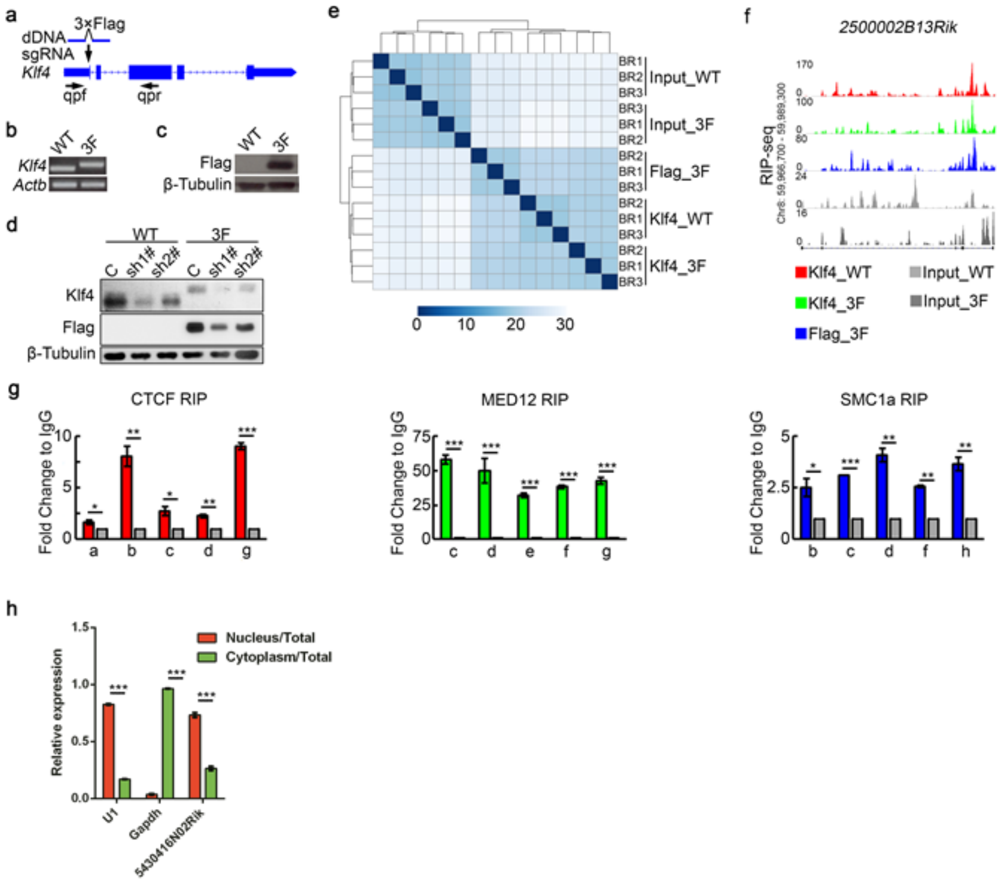

Figure S1. Analysis of RIP-seq profiles.

(a) Schematic illustration of the knock-in strategy at the *Klf4* locus. Blue rectangles represent *Klf4* exons. A donor DNA (dDNA) containing 3′ Flag sequence for *Klf4* N terminal knock-in is shown at the top. A vertical arrow indicates the sgRNA targeting site. Arrows marked with qpf and qpr are primers for *Klf4* RT-PCR.

(b) RT-PCR results demonstrate homozygous knock-in of 3×Flag at the *Klf4* locus. PCR products using qpf and qpr primers show a 66 bp shift in 3F cells, compared with WT cells, due to the 3×Flag knock-in.

(c) Western Blot analysis confirms Flag expression in 3F cells.

(d) Western Blot analysis confirms the correct integration at the *Klf4* locus. WT and 3F cells were transfected with plasmids expressing *Klf4* shRNAs (sh1# and sh2#) as

well as a control vector. Cells were harvested 48 hours after transfection and processed for Western Blot with Klf4, Flag, and β-Tubulin antibodies.

(e) Clustered heatmap using all genes shows the sample-to-sample similarity between RIP-seq profiles. Three biological replicates are shown as BR1, BR2 and BR3. Complete hierarchical clustering is performed, basing on the Euclidean distances between samples.

(f) Representative signals at the *2500002B13Rik* locus from Klf4_WT (red), Klf4_3F (green) and Flag_3F (blue) RIP-seq, as well as input RNAs (grey).

(g) RIP qRT-PCR verifies the binding between CTCF, MED12, SMC1a and *5430416N02Rik* RNA. RIP assays were performed with CTCF antibody, MED12 antibody, SMC1a antibody and IgG antibody in WT ESCs. Eight DNA amplicons (a-h) are illustrated as short black bars in Figure 1e. Data are shown as the mean ± SEM (n=3).*p<0.05, **p<0.01, ***p<0.001.*t*-test.

(h) Cytoplasmic/nuclear fractionation analysis of mESCs shows that 5430416N02Rik is mainly located in the nuclear. U1 and Gapdh serve as positive ctrl of nucleus and cytoplasm, respectively. Data are shown as the mean ± SEM (n=3). ***p<0.001, *t*-test.

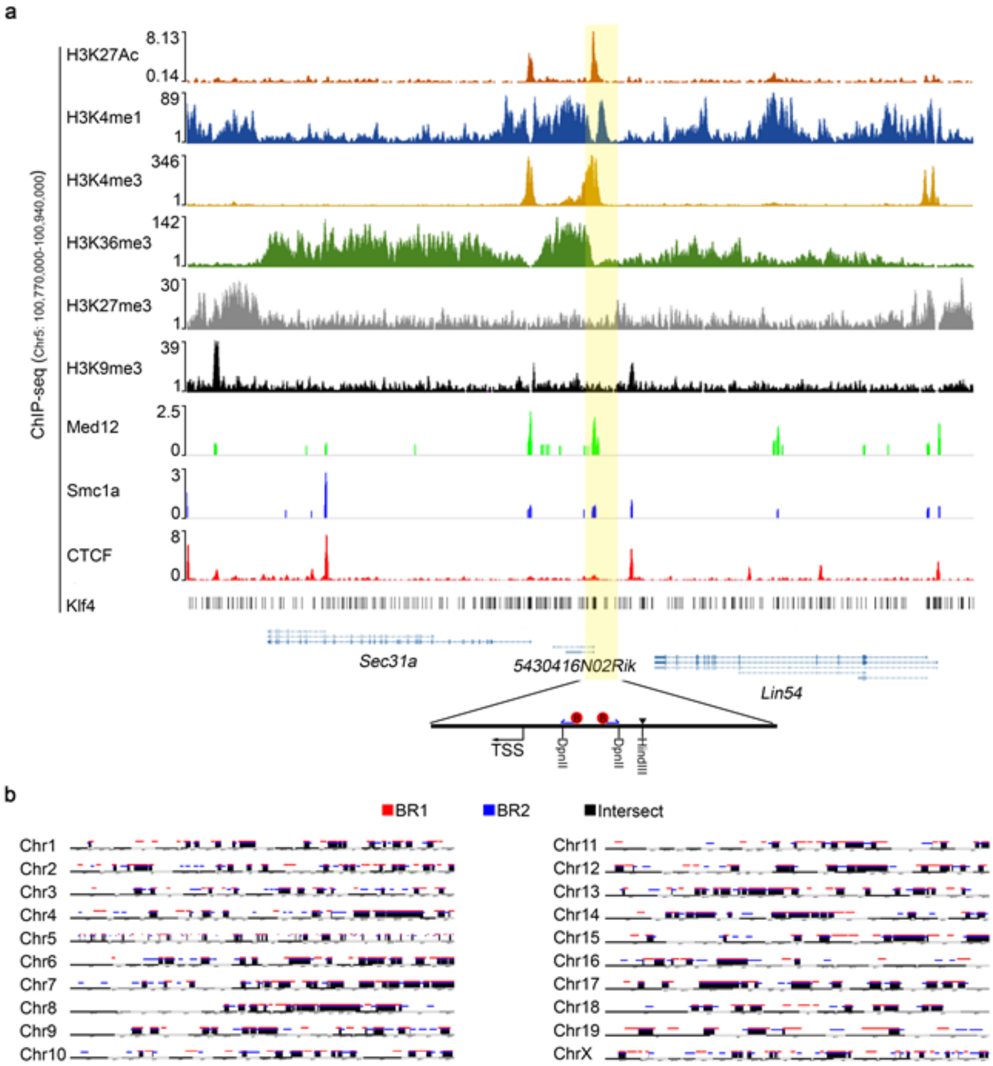

Figure S2. The *5430416N02Rik* promoter is a chromatin interaction hub.

(a) ChIP-seq enrichment patterns for histone modifications and chromatin architectural proteins at the *5430416N02Rik* locus. Tracks assembled in mm9 are displayed in the order of H3K27ac (ENCFF001KFN, ENCODE), H3K4me1 (ENCFF001ZGW, ENCODE), H3K4me3 (ENCFF001ZPH, ENCODE), H3K36me3 (ENCFF001ZHS, ENCODE), H3K27me3 (ENCFF001ZJJ, ENCODE), H3K9me3 (ENCFF001ZHH, ENCODE), Med12 (Pool of GSM560345 and GSM560346, GEO), Smc1a (Pool ofGSM560341 and GSM560342, GEO), CTCF (GSM918748, GEO) and Klf4 (GSM288354, GEO). The *5430416N02Rik* promoter region is zoomed into

and displayed by a bold black line at the bottom. Transcription orientation of *5430416N02Rik* is indicated by a black arrow. Blue arrows with red cycle at DpnII sites represent two biotin-labeled probes for Capture-C. A black triangle at the HindIII site indicates the bait for 4C-seq.

(b) Line plots show regions interacting with the *5430416N02Rik* promoter in each chromosome. Interacting regions identified in the two biological replicates of 4C-seq are shown with red and blue lines, respectively. Black boxes mark overlapped interacting regions from two biological replicates.

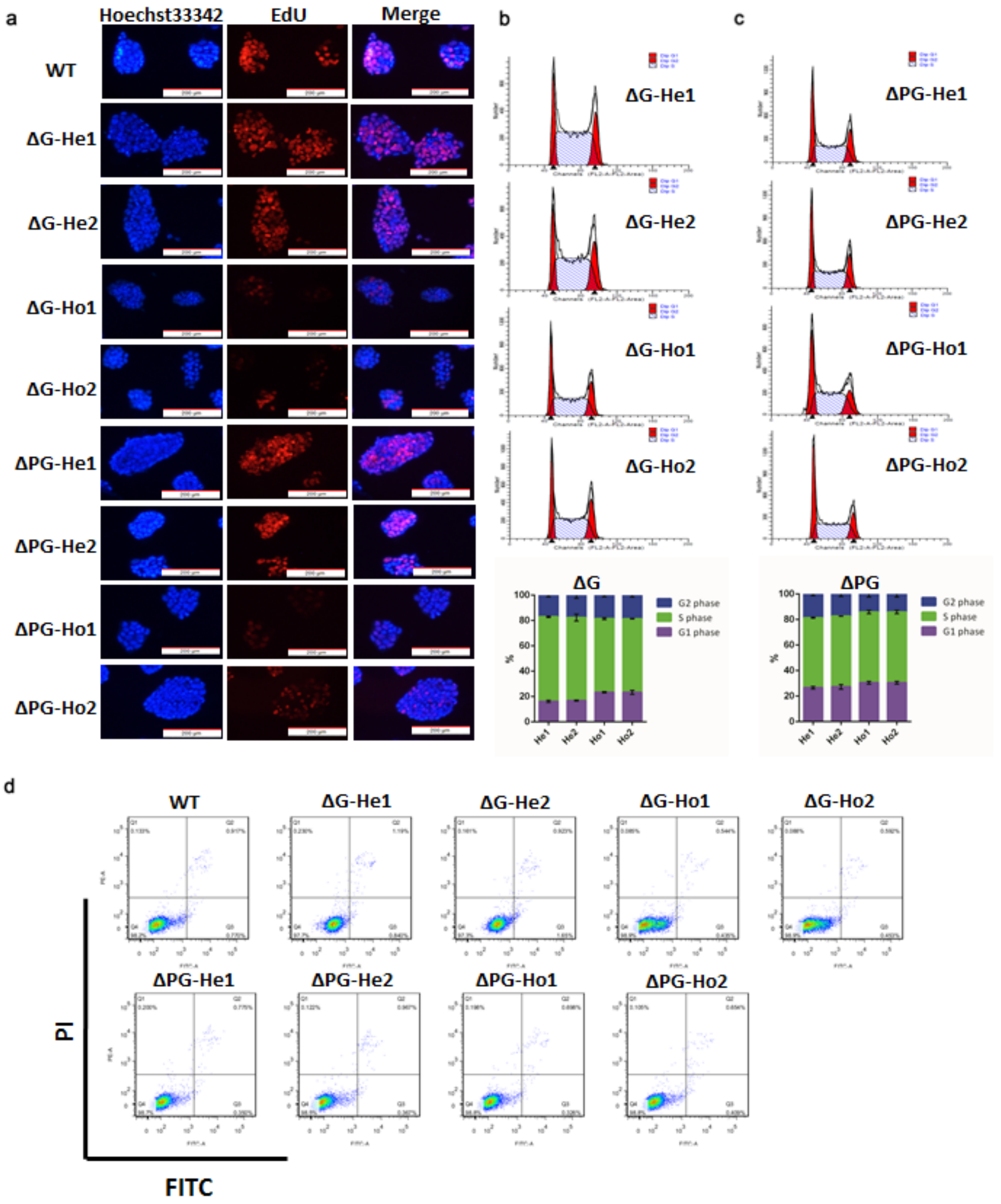

Figure S3. Both homozygous ΔPG and ΔG ESCs show reduced growth rates.

(a) Images of EdU incorporation analysis in WT, ΔG and ΔPG ESCs. Scale bar: 200 µm.

(b-c) Cell cycle analysis and quantification of cell cycle phases in ΔG (b) and ΔPG (c) ESCs.

(d) Cell apoptosis analysis.

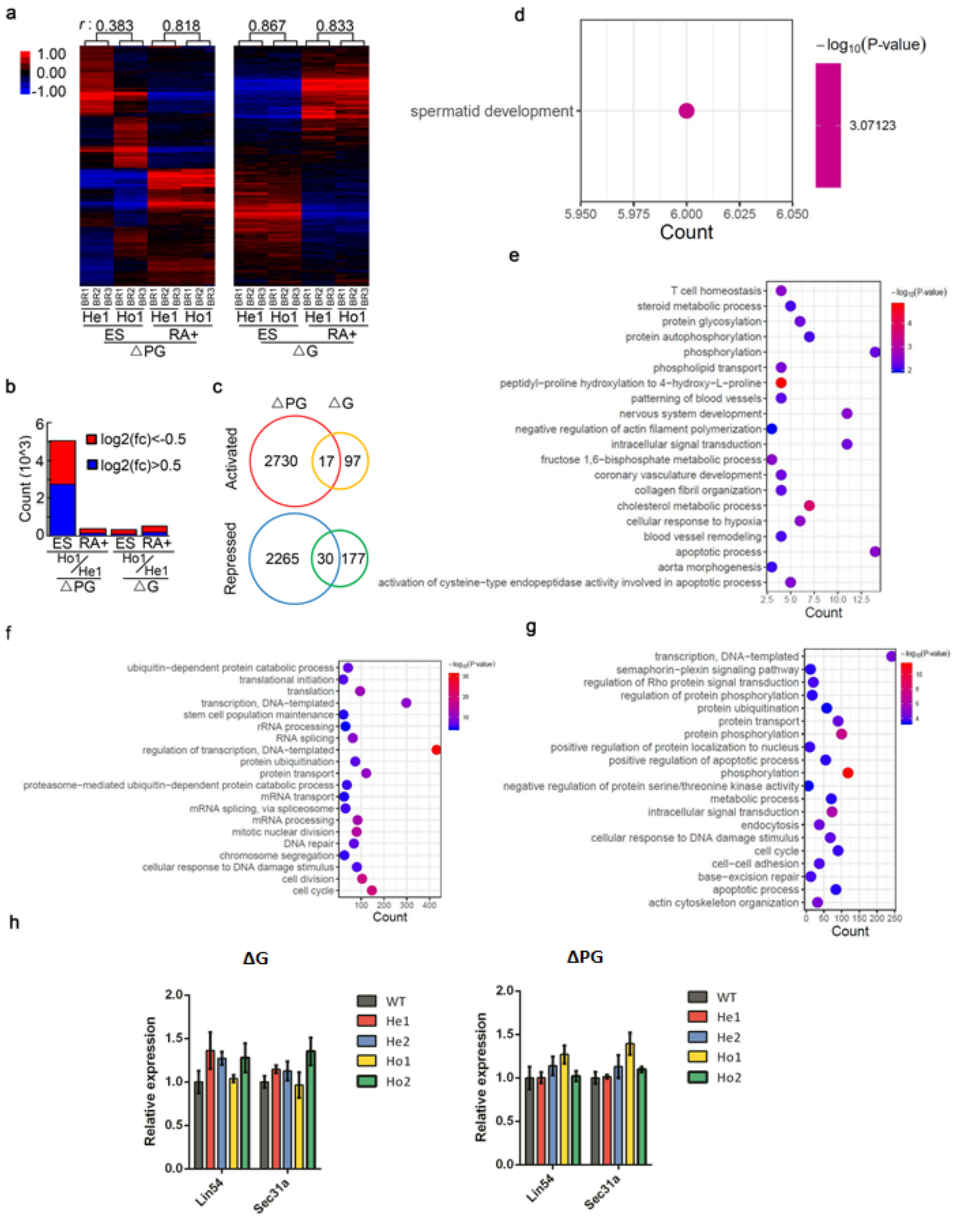

Figure S4. The transcriptional profile is perturbed more in homozygous ΔPG ESCs than in homozygous ΔG ESCs.

(a) Clustering of differentially regulated genes in heterozygous and homozygous ΔPG and ΔG ESCs, as well as RA treated ESCs. ESCs are treated with RA (2 μM) and

DMSO for 48 hours, and harvested for RNA-seq. Three biological replicates are included. Pearson correlation coefficient (*r*) is calculated and shown at the top.

(b) Numbers of activated or repressed genes in homozygous ΔPG and ΔG cells, in comparison to their heterozygous counterparts.

(c) Venn diagrams of activated (top) and repressed genes (bottom) in homozygous ΔPG and ΔG ESCs, in comparison to their heterozygous counterparts.

(d-g) GO analysis of biological progress of genes differentially up-regulated in homozygous ΔG cells (d), down-regulated in homozygous ΔG cells (e), up-regulated in homozygous ΔPG cells (f), down-regulated in homozygous ΔPG cells (g).

(h) Relative mRNA levels of two neighbor genes of 5430416N02Rik in homozygous ΔG and ΔPG and the control cells.

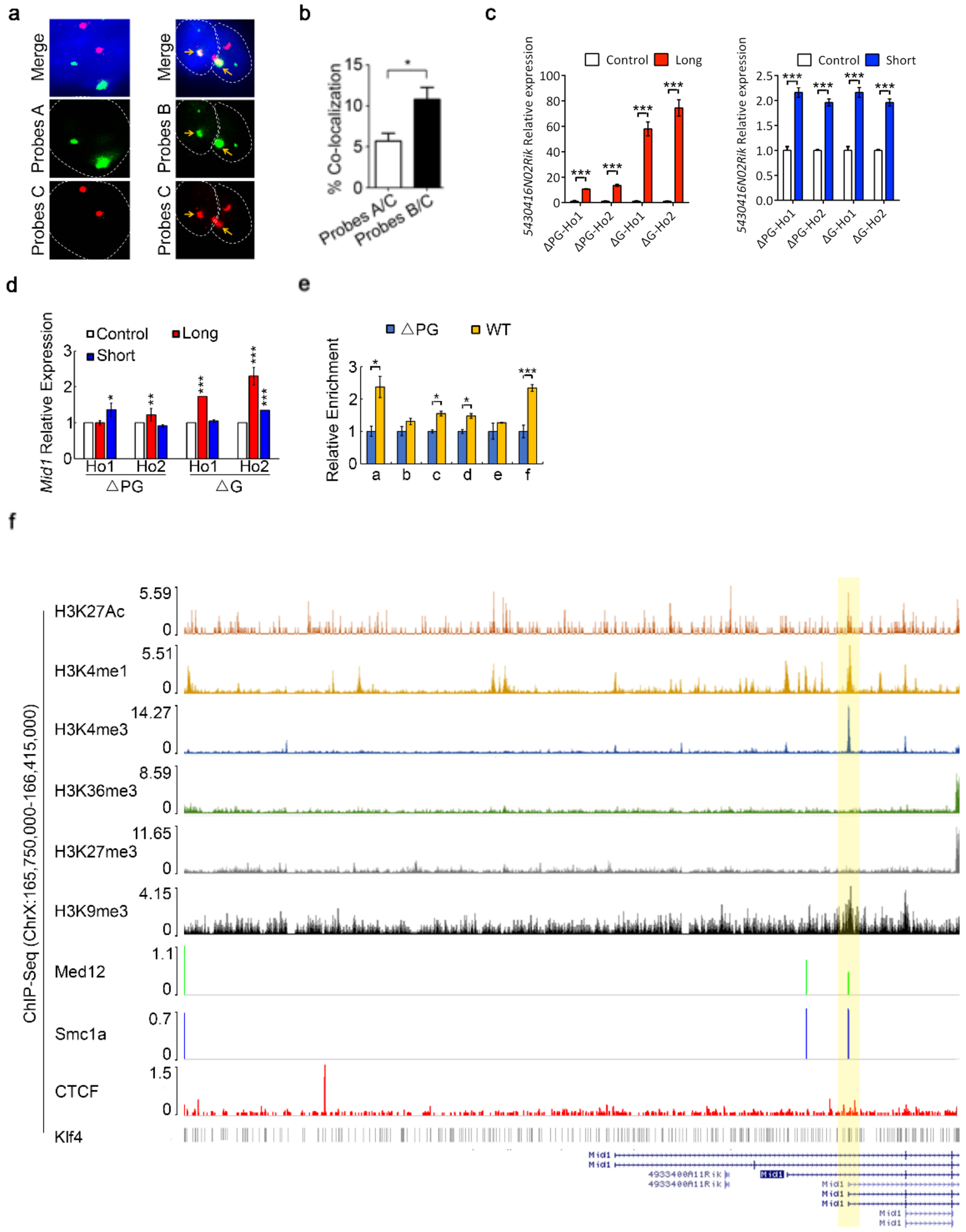

Figure S5. Both *5430416N02Rik* DNA and RNA interact with the *Mid1* locus.

(a) Representative images of DNA FISH show co-localizations of 5430416N02Rik with Col1A1 (left panel) or Mid1 (right panel). Probes A: Col1A1, green, BAC clones (RP23-75B6 and RP23-321D21). Probes B: Mid1, green, BAC clones (RP23-154O12 and RP24-250A6). Probes C: 5430416N02Rik, red, BAC clone (RP24-385C17).

(b) Quantification of the co-localization of 5430416N02Rik with Col1A1 or Mid1

(n=4, more than 50 nuclei were counted for each experiment). Data are shown as the mean ± SEM. *p<0.05,

(c) Relative mRNA levels of two isoform of 5430416N02Rik in homozygous ΔG and ΔPG ESCs that overexpressed two isoform of 5430416N02Rik. Data are shown as the mean ± SEM (n=3). ***p<0.001.*t*-test.

(d) Relative mRNA levels of Mid1 in homozygous ΔG and ΔPG ESCs that overexpressed two isoform of 5430416N02Rik. Data are shown as the mean ± SEM (n=3).*p<0.05, **p<0.01, ***p<0.001.*t*-test.

(e) Following RAP assay, interactions between *5430416N02Rik* RNA and DNA regions of *Mid1* were detected by qRT-PCR. DNA amplicons (a-f) are illustrated as in Figure 4g. Data are shown as the mean ± SEM. *p<0.05, ***p<0.001.*t*-test.

(f) ChIP-seq enrichment patterns for histone modifications and chromatin architectural proteins at the *Mid1* locus. Tracks assembled in mm9 are displayed in the same way of Figure S2a.
