# Supplementary material for: lncRNA 5430416N02Rik Promotes the Proliferation of Mouse Embryonic Stem Cells by Activating Mid1 Expression through 3D Chromatin Architecture
Source: Stem Cell Reports. 2020 Mar 10;14(3):493–505. doi: 10.1016/j.stemcr.2020.02.002 (PMC7066321; doi:10.1016/j.stemcr.2020.02.002)
Supplement: Document S2. Article plus Supplemental Information [file mmc5.pdf]

Stem Cell Reports
Article
OPEN ACCESS

lncRNA *5430416N02Rik* Promotes the Proliferation of Mouse Embryonic Stem Cells by Activating *Mid1* Expression through 3D Chromatin Architecture

Tong Zhao,[1,3] Mingyang Cai,[2,3] Man Liu,[1,3] Guangsong Su,[1] Daniel An,[2] Byoungsan Moon,[2] Guochang Lyu,[2] Yibo Si,[2] Lingyi Chen,[1,*] and Wange Lu[1,*]
[1]State Key Laboratory of Medicinal Chemical Biology, Tianjin Key Laboratory of Protein Sciences and College of Life Sciences, Nankai University, Tianjin 300071, China
[2]Eli and Edythe Broad Center for Regenerative Medicine and Stem Cell Research at USC, Department of Stem Cell Biology and Regenerative Medicine, University of Southern California, Los Angeles, CA 90089, USA
[3]Co-first author
*Correspondence: lingyichen@nankai.edu.cn (L.C.), wangelv@gmail.com (W.L.)
https://doi.org/10.1016/j.stemcr.2020.02.002

SUMMARY

Both 3D chromatin architecture and long non-coding RNAs (lncRNAs) play essential roles in pluripotency maintenance. However, whether lncRNAs are involved in organizing 3D chromatin structure remains largely unexplored. We identified 39 lncRNAs bound by Klf4, among which we further revealed the *5430416N02Rik* promoter is a chromatin interaction hub. Knockout of the *5430416N02Rik* locus reduces the proliferation rate of embryonic stem cells (ESCs). Moreover, deleting both the promoter and the gene body of *5430416N02Rik* causes a more severe proliferation defect and has a more profound impact on the transcriptome than deleting the gene body alone. The reduced proliferation of the *5430416N02Rik* locus knockout ESCs is mainly due to the downregulation of *Mid1*, the expression of which requires the inter-chromosomal interaction between *Mid1* and *5430416N02Rik* loci. In summary, our data demonstrated that the lncRNA *5430416N02Rik* gene locus maintains the fast proliferation of ESCs by activating the expression of *Mid1* through chromatin interaction.

INTRODUCTION

Embryonic stem cells (ESCs), derived from the inner cell mass of pre-implantation blastocysts, are able to self-renew indefinitely, while maintaining the differentiation potential into all types of cells in the body (Evans and Kaufman, 1981). Understanding the molecular mechanisms of pluripotency maintenance is beneficial for the potential application ESCs in regenerative medicine. It has become clear that 3D chromatin architecture plays an essential role in the establishment and maintenance of pluripotency (Apostolou et al., 2013; Wei et al., 2013a, 2013b; Zhang et al., 2013). Genomes are organized into topologically associated domains (TADs), which are conserved in different cell types and across species (Dixon et al., 2012; Ji et al., 2016). Yet, extensive chromatin reorganization, altering chromatin interactions both within and between TADs, occurs during lineage specification of ESCs (Dixon et al., 2015; Phillips-Cremins et al., 2013). More importantly, unique pluripotency-specific chromatin interactomes around the key pluripotency gene loci, *Nanog* and *Oct4*, are maintained in ESCs, and are functionally important for pluripotency (Apostolou et al., 2013; Wei et al., 2013a).

Chromatin architectural proteins (CAPs), such as CTCF, Cohesin, and Mediator, regulate and stabilize chromatin interactions (Dixon et al., 2012; Handoko et al., 2011; Kagey et al., 2010; Tang et al., 2015). In addition, Klf4, a pluripotency transcription factor, together with Cohesin and Mediator, organizes long-range chromosomal interactions at the *Oct4* locus in mouse ESCs (mESCs) (Wei et al., 2013a). However, it is difficult for these relatively small and globular CAPs to bring relatively bulky chromatin regions close and stabilize the chromatin interaction, even though protein complexes are formed by CAPs. Due to the flexible linear shape, long non-coding RNAs (lncRNAs) might serve as scaffold molecules to assist CAPs in organizing chromatin architecture. For example, lncRNA *Firre*, interacts with hnRNPU to maintain the interactions of its own gene locus with five distinct trans-chromosomal loci (Hacisuleyman et al., 2014). In addition, lncRNAs have been shown to be involved in the self-renewal and differentiation of ESCs, as well as somatic cell reprogramming (Guttman et al., 2011; Loewer et al., 2010; Yin et al., 2015). Nevertheless, whether lncRNAs contribute to pluripotency maintenance through organizing 3D chromatin architecture remains elusive.

In this study, we investigated the role of lncRNA locus in organizing 3D chromatin architecture essential for pluripotency maintenance. Through RNA-binding protein immunoprecipitation coupled with sequencing (RIP-seq) experiments, we first identified 39 Klf4-bound lncRNAs. Taking account of known functional lncRNAs in maintaining and inducing pluripotency, we further studied the lncRNA *5430416N02Rik*. 4C and Capture-C experiments

This is an open access article under the CC BY license (http://creativecommons.org/licenses/by/4.0/).

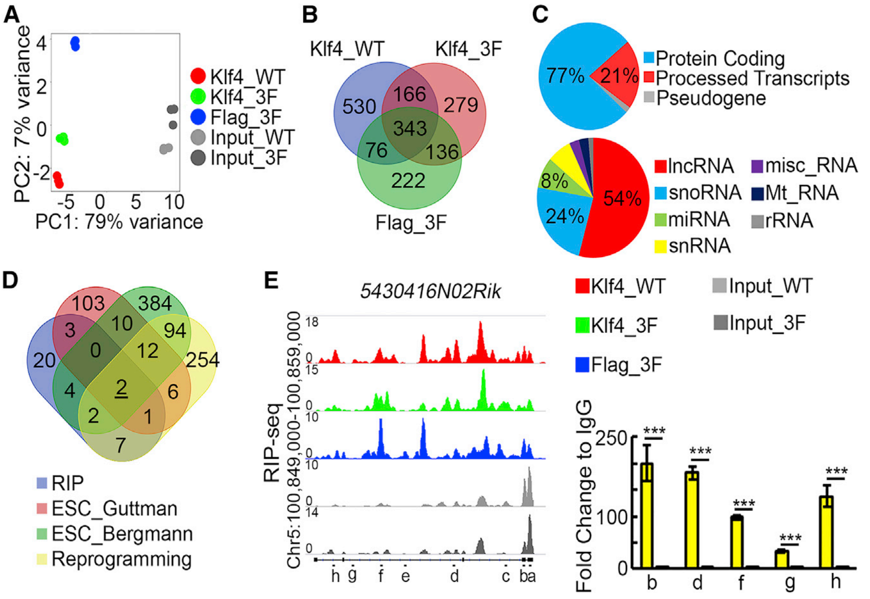

Figure 1. Identification of Potential CAP-Associated RNA

(A) Principal-component analysis of RIP-seq results. Three biological replicates were performed for each RIP-seq experiment.

(B) High-confidence Klf4-bound RNAs identified by overlapping significantly enriched RNAs in three RIP-seq experiments. The detailed information of these RNAs is listed in Table S1.

(C) Classification of Klf4-bound RNAs (upper panel) and processed transcripts (lower panel).

(D) Comparison of Klf4-bound lncRNAs (RIP), lncRNAs regulating ESC transcriptome (ESC_Guttman and ESC_Bergmann), and lncRNAs activated during reprogramming (Reprogramming). The detailed information of these lncRNAs is listed in Table S1.

(E) Left panel shows representative signals at the *5430416N02Rik* locus from Klf4_WT (red), Klf4_3F (green), and Flag_3F (blue) RIP-seq, as well as input RNAs (gray). Eight DNA amplicons (a–h) are illustrated as short black bars. Right panel shows the result of RIP qRT-PCR with Klf4 antibody and IgG control. Three biological replicates per sample were assayed. Data are shown as the mean ± SEM (n = 3). ***$p < 0.001$, t test.

coupled with sequencing demonstrated that the promoter of *5430416N02Rik* is a chromatin interaction hub. Moreover, knocking out both the promoter and the gene body of *5430416N02Rik* affects more genes and causes a more severe proliferation defect in ESCs, in comparison with knockout of the *5430416N02Rik* gene only, demonstrating the additional function of the *5430416N02Rik* promoter other than driving the transcription of *5430416N02Rik*. Mechanistically, knockout of the *5430416N02Rik* locus impairs the inter-chromosomal interaction between *5430416N02Rik* and *Mid1* loci, leads to downregulated *Mid1* transcription, and consequently reduces the proliferation rate of ESCs. Our work expanded the knowledge of how lncRNAs gene loci regulate pluripotency by organizing 3D chromatin architecture in ESCs.

RESULTS

Identification of CAP-Associated lncRNAs

We demonstrated previously that Klf4, a pluripotency transcription factor, together with Cohesin and Mediator, organizes long-range chromosomal interactions at the *Oct4* locus in mESCs (Wei et al., 2013a). To test the possibility that lncRNAs serve as scaffold molecules, and collaborate with Klf4 and other CAPs to form large RNA/protein complexes and organize 3D chromatin architecture, RIP-seq was carried out to detect whether Klf4 binds to any lncRNAs. The first RIP (Klf4_WT) was performed with a Klf4 antibody to pull-down endogenous Klf4 in wild-type (WT) ESCs. To circumvent the specificity issue of the Klf4 antibody, two additional RIP experiments (Klf4_3F and Flag_3F) were conducted in 3F ESCs, in which 3×Flag tag is fused to the N terminus of endogenous Klf4 (Figures S1A–S1D), with Klf4 and Flag antibodies, respectively. Both principal-component analysis (PCA) and sample-to-sample distance analysis demonstrate the robust consistency of three biological replicates and the similarities among these three RIP samples, in comparison with input RNA samples (Figures 1A and S1E). Analyzing each RIP-seq profile with the DESeq2 pipeline, using the corresponding input profile as the background, 1,115, 924, and 777 significantly enriched RNAs were identified in Klf4_WT, Klf4_3F, and Flag_3F profiles, respectively (Table S1). Overlapping the three lists of significantly enriched RNAs allowed us to identify 343 Klf4-bound RNA with high confidence (Figure 1B; Table S1). Seventy-seven percent of these Klf4-bound RNAs encode proteins, and 72 RNAs (21%) belong to the processed transcripts lacking open reading frame. 39 out of the 72 non-coding RNAs (54%) are lncRNAs (Figure 1C). These 39 Klf4 bound lncRNAs are candidates for CAP-associated RNA.

Proper long-range chromatin interactions are essential for the self-renewal of ESCs and somatic cell reprogramming. Thus, we expected that CAP-associated lncRNAs in ESCs should play roles in pluripotency maintenance and reprogramming. Previous studies have identified lncRNAs regulating ESC transcriptome (Bergmann et al., 2015; Guttman et al., 2011), and lncRNAs activated during reprogramming (Kim et al., 2015). Comparison of the 39 Klf4 bound

lncRNAs to these 3 groups of lncRNAs identified two lncRNAs, *5430416N02Rik* (also known as *Gm3514* and *Adapt33*), and *2500002B13Rik*, shared by four lists (Figures 1D, 1E, and S1F; Table S1). Hence, these two lncRNAs are most likely CAP RNAs.

Among these two CAP RNA candidates, *5430416N02Rik* RNA is also bound by CTCF protein, a well-known insulator binding protein involved in chromatin interactions (Kung et al., 2015), further suggesting *5430416N02Rik*'s role as a CAP-associated RNA. RIP coupled with qRT-PCR was performed to validate the RIP-seq result, and revealed strong binding of Klf4 to *5430416N02Rik* RNA (Figure 1E). It has been shown that *5430416N02Rik* is a multiple stress-responsive riboregulator (Wang et al., 1996, 2003). Cytoplasmic/nuclear fractionation assay shows that the *5430416N02Rik* transcript is abundant in the nucleus of mESCs (Figure S1H). Yet, the function of *5430416N02Rik* in ESCs is not well characterized. So we focused on investigating the function of *5430416N02Rik* in ESCs, especially its role in 3D chromatin architecture.

Functions of *5430416N02Rik* in Regulating Chromatin Interaction

Unlike proteins, lncRNAs often exert their function immediately following or even during their transcription without processing or translocation (Quinn and Chang, 2016). If *5430416N02Rik* functions in *cis* to regulate 3D chromatin architecture, its gene locus should be an organizing hub for chromatin interactions. We investigated the histone codes as well as the occupations of architecture proteins at the *5430416N02Rik* gene locus, considering the tight relationship between genome-wide interactions and activating chromatin marks (Huang et al., 2015). There is a good correlation of active histone marks (H3K27ac, H3K4me1, and H3K4me3) and CAPs (Med12, Smc1a, and Klf4) binding signals at the *5430416N02Rik* promoter region (Figure S2A), implying that the promoter of *5430416N02Rik* may have interactions with other chromatin regions in ESCs.

To demonstrate that the promoter region of *5430416N02Rik* is a chromatin interaction hub, we performed circular chromosome conformation capture followed by high throughput sequencing (4C-seq), with a bait located about 1 kb upstream the transcription starting site (TSS) of *5430416N02Rik* using HindIII (Figure S2A). 339 and 341 significant *5430416N02Rik*-interacting regions were identified in two independent biological replicates of 4C-seq experiments (Table S2). Overlapping the identified regions from two biological replicates revealed 300 *5430416N02Rik*-interacting regions with high confidence (Figure S2B). Further analysis revealed that 12,717 genes are located in the 300 *5430416N02Rik*-interacting regions (Table S2). Thus, our 4C-seq and previous chromatin immunoprecipitation sequencing (ChIP-seq) results collaboratively suggest that the *5430416N02Rik* promoter is a chromatin interaction hub.

To further prove the function of the *5430416N02Rik* locus in regulating chromatin interaction, we constructed heterozygous and homozygous ΔG ESC lines, in which the gene body of *5430416N02Rik* is replaced by a "GFP-IRES-Puro" cassette (Figures 2A and 2B), and performed Capture-C experiment followed by sequencing, using heterozygous and homozygous ΔG ESC lines. The bait for the Capture-C experiment is located just nearby the bait we used in 4C libraries. We detected 2,423 and 2,820 *5430416N02Rik*-interacting sites in the replicates of heterozygous and homozygous ΔG ESCs, respectively (Table S2). These *5430416N02Rik*-interacting sites are distributed along all of the chromosomes, while about half of the interactions are within chromosome 5 where *5430416N02Rik* is located (Figure 2C). Most importantly, we identified 1730 differential *5430416N02Rik*-interacting sites between heterozygous and homozygous ΔG ESCs, which are widely spread out through the genome (Figure 2D; Table S2). It provides evidence that heterozygous and homozygous ΔG ESCs have very different chromatin interactions.

Both the Promoter and the Gene Locus of *5430416N02Rik* Play Important Roles in ESC Proliferation

To investigate the biological function of the *5430416N02Rik* locus, we characterized the phenotype of heterozygous and homozygous ΔG ESCs. Given the importance of the *5430416N02Rik* promoter in mediating chromatin interactions, we also constructed ΔPG ESC lines, in which the gene body and the promoter of *5430416N02Rik* are both knocked out (Figure 2A). Two independent heterozygous clones (He1 and He2) and homozygous clones (Ho1 and Ho2) of ΔG and ΔPG ESCs are used for phenotype characterization (Figures 2A and 2B). Notably, homozygous ΔG and ΔPG ESCs grow slower than WT and heterozygous ESCs. Intriguingly, homozygous ΔPG ESCs display more severe proliferation deficiency than homozygous ΔG ESCs (Figure 3A), indicating an additional function of the *5430416N02Rik* promoter other than driving the expression of *5430416N02Rik*. It is consistent with our 4C-seq result that the *5430416N02Rik* promoter mediates long-range chromatin interactions. To further investigate the effect of 5430416N02Rik on proliferation of mESCs, we performed the 5-ethynyl-20-deoxyuridine (EdU) incorporation assay. The results showed that homozygous ΔG and ΔPG ESCs exhibited lower EdU incorporation than the control cells (Figure S3A). We also performed cell-cycle analysis of ΔG and ΔPG mESCs using propidium iodide staining. As expected, homozygous ΔG and ΔPG ESCs both had a higher proportion G1 phase cells,

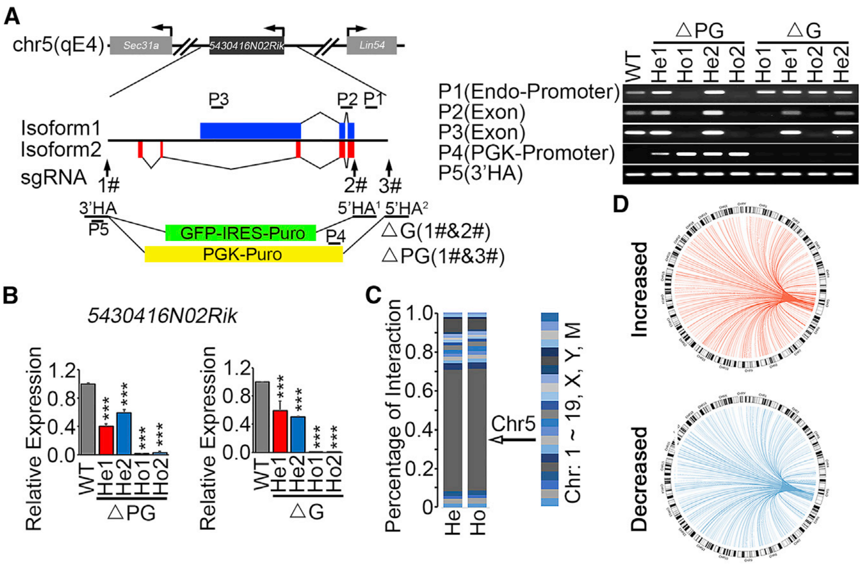

Figure 2. *5430416N02Rik* RNA Is Required for the Chromatin Interactions at Its Promoter

(A) Schematic illustration of knockout strategy at the *5430416N02Rik* locus. Two isoforms of *5430416N02Rik* are shown with blue and red boxes, respectively. The gene body and the promoter (~2 kb upstream of TSS) of *5430416N02Rik* are replaced by a PGK-puro cassette, resulting in homozygous (Ho) or heterozygous (He) ΔPG ESCs. In ΔG cells, only the gene body is replaced by a GFP-IRES-Puro cassette. He1&2 and Ho1&2 are two independent heterozygous and homozygous clones, respectively. Vertical arrows mark targeting sites of three sgRNAs. Homology arms (3′ HA, and 5′ HA[1] and 5′ HA[2]) are shown in bold. Short black lines, P1 to P5, marks PCR amplicon for genotyping. The bottom panel shows the genotyping results.

(B) *5430416N02Rik* expression levels in heterozygous and homozygous ΔPG and ΔG ESCs, as well as WT ESCs, detected by qRT-PCR. Three biological replicates per sample were assayed. Data are shown as the mean ± SEM (n = 3). ***$p < 0.001$, t test.

(C) Numbers of interacting sites with the *5430416N02Rik* promoter in individual chromosomes identified by Capture-C-seq are shown in 100% stacked column.

(D) Circos plots of differential *5430416N02Rik*-interacting sites in homozygous ΔG ESCs, compared with heterozygous ΔG ESCs, identified by Capture-C-seq. Two biological replicates were performed for each Capture-C experiment.

suggesting ΔG and ΔPG causes G1 phase cell-cycle arrest and a severe proliferation defect (Figures S3B and S3C). To determine whether ΔG and ΔPG affect mESCs survival, we used Annexin V-FITC staining to detect apoptosis. We did not observe significant changes in apoptosis in homozygous ΔG and ΔPG ESCs compared with the control (Figure S3D). Besides, the slow proliferation rates of homozygous ΔG and ΔPG ESCs are not due to reduced expression levels of pluripotency genes (Figures 3B and 3C). When induced to differentiate by retinoic acid, homozygous ΔPG ESCs differentiate slightly more toward endoderm lineage, and less toward ectoderm, while homozygous ΔG does not affect the differentiation of ESCs (Figures 3D and 3E). In embryoid body (EB) differentiation experiments, EBs from homozygous ΔPG ESCs are smaller than those formed by heterozygous ΔPG ESCs (Figure 3F). In contrast, EBs formed by heterozygous and homozygous ΔG ESCs are about the same size (Figure 3G). The more severe differentiation defects of homozygous ΔPG ESCs further suggest additional function of the *5430416N02Rik* promoter, except for the function as a promoter.

5430416N02Rik Regulates the Proliferation of ESCs through *Mid1*

To identify the downstream target genes of the *5430416N02Rik* locus involved in ESC proliferation, RNA sequencing (RNA-seq) was carried out to analyze the transcriptional profiles of heterozygous and homozygous ΔG and ΔPG ESCs, under undifferentiated and differentiated conditions. As expected, in undifferentiated ESCs, homozygous ΔPG affects the transcriptome more than homozygous ΔG (Figure S4A and S4B). Compared with their heterozygous counterparts, homozygous ΔPG activates 2,747 genes and represses 2,295 genes, while ΔG leads to 114 upregulated genes and 207 downregulated genes (Figure S4B; Table S3). Among them, 17 activated genes and 30 repressed genes are shared by homozygous ΔPG and ΔG ESCs (Figure S4C). Gene ontology analysis of biological processes showed that many downregulated genes in ΔPG ESCs were related to cell cycle, which is consistent with our results that homozygous ΔG and ΔPG ESCs grow slower than WT and heterozygous ESCs (Figures S4D–S4G). LncRNA was known to play important roles in regulating expression of neighbor genes. We examined the expression of two neighbor genes of 5430416N02Rik, Lin54 and Sec31a, in homozygous KO cells and the control cells and found that the expressions of these two genes were not affected (Figure S4H).

Because both homozygous ΔPG and ΔG ESCs show reduced growth rates, differentially expressed genes shared by homozygous ΔPG and ΔG ESCs likely account for the slow growth. Among these 47 differentially expressed genes, *Mid1*, located at chromosome X, has been reported as an important factor regulating ESC proliferation through the MID1-PP2A-CDC25B-CDK1 signaling pathway (Ko et al., 2016). We first validated the RNA-seq data by qRT-PCR,

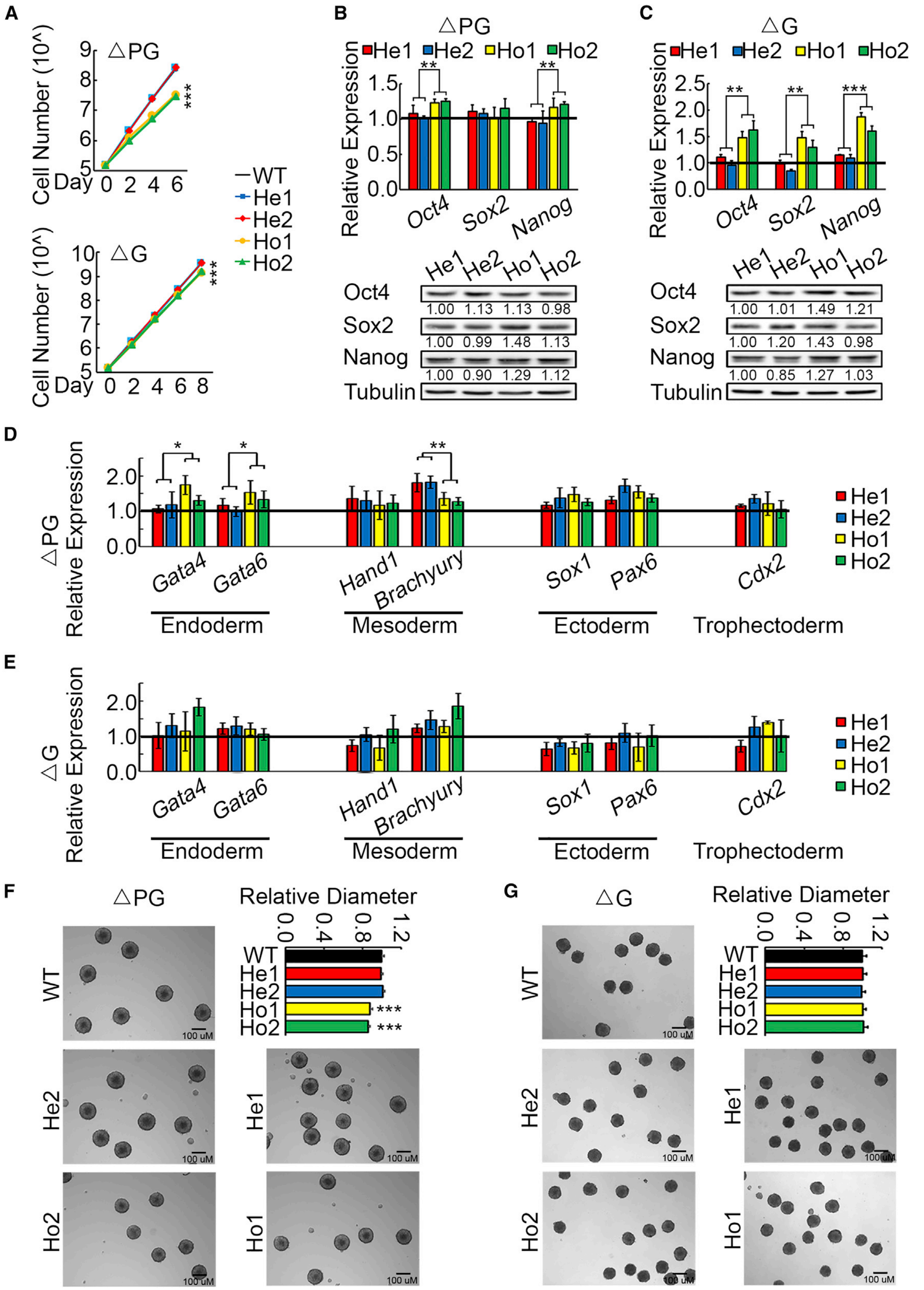

(legend on next page)

and confirmed that the expression level of *Mid1* is reduced in both homozygous ΔPG and ΔG ESCs, which is consistent with the slow growth rate in both homozygous ΔPG ESCs and ΔG ESCs (Figure 4A). Similar to the phenotype of homozygous ΔG and ΔPG ESCs, ESCs with *Mid1* knockdown also grow slower (Figures 4B and 4C). Conversely, overexpression of *Mid1* leads to larger colonies in colony-forming assay (Figures 4D–4F). Most importantly, *Mid1* overexpression rescued the reduced colony size of homozygous ΔG and ΔPG ESCs (Figures 4E and 4F), indicating that *Mid1* is a downstream target of *5430416N02Rik* in regulating ESC proliferation.

The Interaction between *5430416N02Rik* and *Mid1* Loci Activates *Mid1* Expression

Our 4C-seq data revealed that the *5430416N02Rik* promoter interacts with the *Mid1* promoter (Table S2). Moreover, Capture-C data further demonstrated that the interaction between *5430416N02Rik* and *Mid1* is perturbed in homozygous ΔG ESCs (Table S2). We performed 3C experiments to validate our 4C-seq and Capture-C result, and identified three sites (a, d, and e) in the *Mid1* locus, interacting with the *5430416N02Rik* promoter. More importantly, interaction frequencies at the d and e sites of the *Mid1* locus significantly decreased in homozygous ΔG ESCs (Figure 4G). DNA fluorescence *in situ* hybridization (FISH) experiments further validated the interaction between *5430416N02Rik* and *Mid1* loci (Figures S5A and S5B).To further elucidate the function of 5430416N02Rik RNA in mediating chromatin interaction, we tried to knock down the 5430416N02Rik expression with small hairpin RNA (shRNA) and small interfering RNA, but neither of them worked. We next performed rescue experiment in ΔG and ΔPG KO ESCs. Overexpression of the long isoform of 5430416N02Rik could partially rescue Mid1 expression (Figures S5C and S5D). Purification DNA by RNA antisense purification (RAP) demonstrated that *5430416N02Rik* RNA associates with the *Mid1* locus (Figure S5E). ChIP-seq data also revealed that Med12, Smc1a, CTCF, and Klf4 bind to the *Mid1* locus, implying that these CAPs are involved in maintaining the chromatin interactions at the *Mid1* locus (Figure S5F). These data suggest that the *5430416N02Rik* locus can interact with the Mid1 locus. *5430416N02Rik* transcripts might be involved in the interaction between *5430416N02Rik* and *Mid1* loci, which promotes the transcription of *Mid1* (Figure 4H).

DISCUSSION

RNA is an integral component of chromatin, and regulates the chromatin structure (Mondal et al., 2010). Yet, whether and how lncRNAs contribute to 3D chromatin organization remain elusive. Here, we aimed to identify lncRNAs involved in maintaining ESC-specific 3D chromatin structure. We rationalized that lncRNAs bound by Klf4, a pluripotency transcription factor contributing to long-range chromatin interactions in ESCs, are likely serving as scaffold molecules to form RNA/protein complexes and stabilize chromatin interactions. Thus, we performed RIP-seq experiment and identified 39 Klf4-bound lncRNAs. Combined with existing functional data of lncRNAs in ESCs, we narrowed down to the lncRNA *5430416N02Rik*, and further demonstrated the role of the *5430416N02Rik* locus in regulating 3D chromatin structure. First, the *5430416N02Rik* promoter is a chromatin interaction hub, as revealed by 4C-seq and Capture-C. Second, knockout of the *5430416N02Rik* gene (ΔG) leads to a global change of the chromatin interaction profile of the *5430416N02Rik* promoter, indicating the essential function of the *5430416N02Rik* gene locus in maintaining 3D chromatin structure. Third, homozygous ΔPG ESCs show more severe defects in proliferation and differentiation, than homozygous ΔG ESCs. It suggests that the *5430416N02Rik* promoter has additional functions other than driving the transcription of *5430416N02Rik*. The additional function of the *5430416N02Rik* promoter is likely to exert long-range regulatory effect through inter- and intra-chromosomal interactions. Indeed, we demonstrated that the interaction between *5430416N02Rik* and *Mid1* loci is required for the expression of *Mid1*, which in turn promotes the fast proliferation of ESCs.

LncRNA as an important regulator, is involved in multiple functions, including cell growth and pluripotency in mESCs. For example, lncRNA Gas5 is required for the maintenance of mESCs self-renewal and proliferation through

Figure 3. Homozygous ΔPG ESCs Display a More Severe Proliferation Defect than Homozygous ΔG ESCs
(A) Proliferation assays for ΔPG, ΔG, and WT ESCs. The Y axis is log scale of cell number. Three biological replicates per sample were assayed. Data are shown as the mean ± SEM (n = 3). ***p < 0.001; one-way ANOVA analysis.
(B and C) Expression levels of pluripotency genes in ΔPG (B) and ΔG (C) ESCs detected by qRT-PCR and western Blot. Three biological replicates per sample were assayed. Data are shown as the mean ± SEM (n = 3). **p < 0.01, ***p < 0.001; one-way ANOVA analysis.
(D and E) Expression levels of differentiation genes in differentiated ΔPG (D) and ΔG (E) ESCs detected by qRT-PCR. ESCs are treated with retinoic acid (2 μM) for 4 days to differentiate. Three biological replicates per sample were assayed. Data are shown as the mean ± SEM (n = 3). *p < 0.05, **p < 0.01. Contrast in one-way ANOVA analysis was carried out (A–E).
(F and G) Relative size of EBs formed by ΔPG (F), ΔG (G), and WT ESCs. Three biological replicates per sample were assayed. Data are shown as the mean ± SEM, ***p < 0.001; one-way ANOVA analysis. Scale bars, 100 μm.

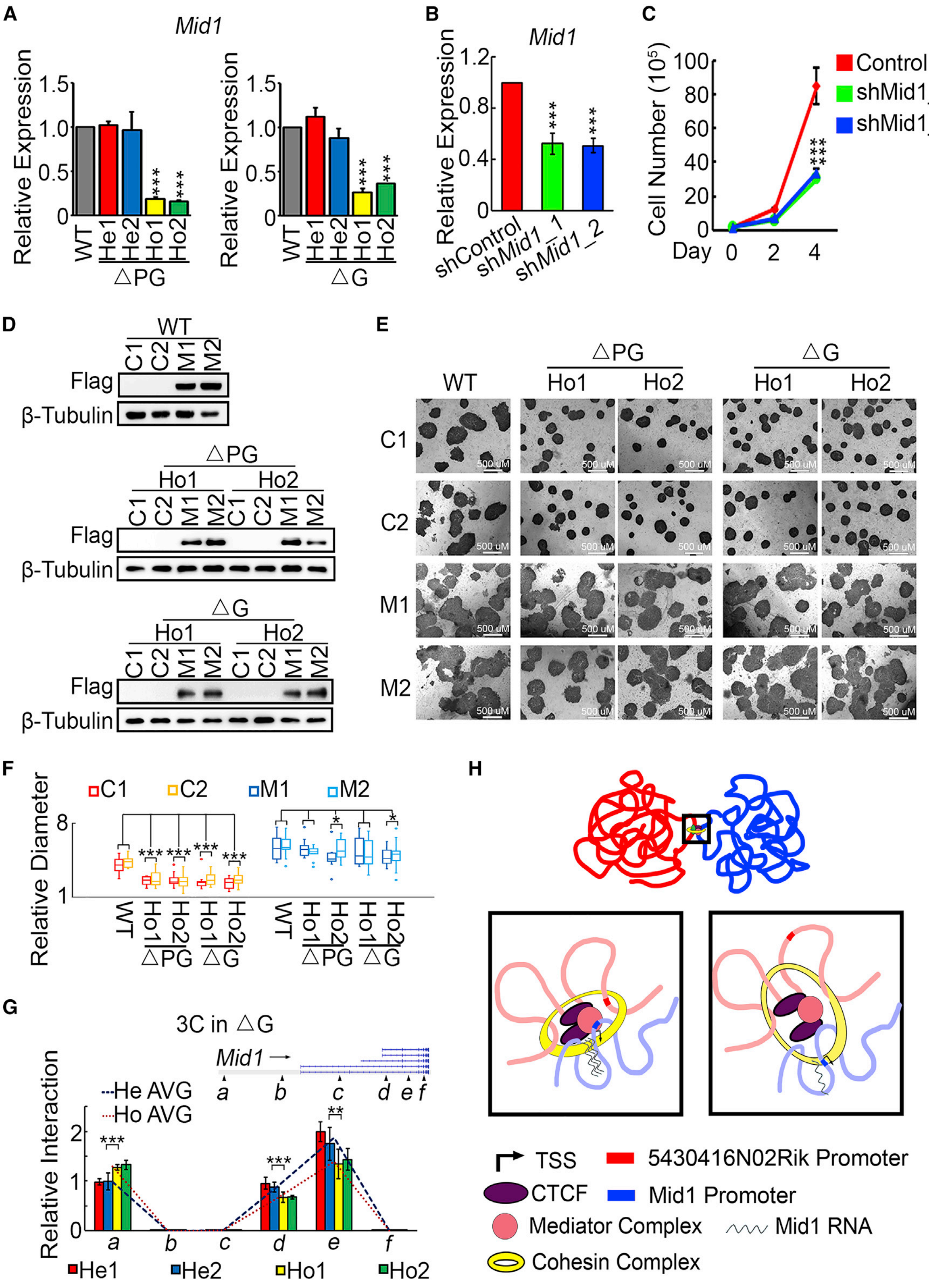

Figure 4. The Chromatin Interaction between *Mid1* and *5430416N02Rik* Loci Activates *Mid1* Expression, and Consequently Promotes ESC Proliferation

(A) *Mid1* expression levels in heterozygous and homozygous ΔPG and ΔG ESCs, as well as WT ESCs, detected by qRT-PCR. Three biological replicates per sample were assayed. Data are shown as the mean ± SEM (n = 3). ***p < 0.001, t test.

(legend continued on next page)

interactions with pluripotent transcriptional factors and DNA demethylation regulators (Tu et al., 2018). LncRNA Trincr1 binds TRIM71 to inhibit its SHCBP1 stabilizing activity, and then sensitizes mESCs to depend on Trim71 for rapid proliferation (Li et al., 2019). Our studies reveal that the lncRNA 5430416N02Rik locus regulates Mid1 expression through 3D chromatin organization, and 5430416N02Rik RNA may participate in mediating this chromatin interaction.

How *5430416N02Rik* RNA collaborates with other CAPs to maintain 3D chromatin architecture remains largely unclear. It has been shown that Klf4 and CTCF bind to *5430416N02Rik* RNA (Kung et al., 2015) (Figure 1). It is likely that more proteins, together with Klf4 and CTCF, bind to *5430416N02Rik* RNA, and stabilize chromatin interactions. Therefore, characterization of the RNA/protein complex containing *5430416N02Rik* RNA will shed light on the molecular mechanism of 3D chromatin organization. *5430416N02Rik* RNA may function as a scaffold in the RNA/protein complex mediating the interaction between the *5430416N02Rik* locus and *Mid1* loci, which needs further research. It is interesting to elucidate the protein components and conformation change of the RNA/protein complex upon *5430416N02Rik* knockout. Alternatively, it is possible that in homozygous ΔG ESCs, other lncRNA might replace *5430416N02Rik* RNA, and function as the scaffold in the RNA/protein complex.

EXPERIMENTAL PROCEDURES

Cell Culture

mESCs D3 were grown in culture dishes coated with 0.1% gelatin (Sigma) in growth medium consisting of 85% DMEM (Gibco), 15% fetal bovine serum (HyClone), 2 mM L-glutamine, 5,000 units/mL penicillin and streptomycin, 0.1 mM non-essential amino acids (Invitrogen), 0.1 mM 2-mercaptoethanol (Sigma), 1 mM sodium pyruvate (Thermo), and 1,000 units/mL leukemia inhibitory factor (LIF) (ESGRO). mESCs D3 were grown without feeder. The medium was replaced every 2–3 days. Cells were maintained at 37°C in a 5% CO_2 incubator. For the pluripotency genes analysis, ESCs were plated at an appropriate density and cultured for 2 days, and then collected for RNA extraction and qPCR.

EB Differentiation

To form EBs, ESCs were cultured in 30 μL hanging drops (1,000 cells/drop, ESC medium without LIF), and EBs were collected on day 4.

Cell-Cycle Analysis

The cell cycle was analyzed using the Cell Cycle and Apoptosis Analysis Kit (Beyotime, C1052) in accordance with the manufacturer's instructions.

Flow Cytometry Analysis of Cell Apoptosis

Cell apoptosis was analyzed using an Annexin V-FITC Apoptosis Detection Kit (Beyotime, C1062L) in accordance with the manufacturer's instructions. Untreated cells were used as a negative control and for gating.

EdU Incorporation Analysis

EdU incorporation was performed using the EdU Detection Kit (Ribobio, C10310-1) in accordance with the manufacturer's instructions. In brief, mESCs were plated at an appropriate density in 12-well plates. Twenty-four hours later, EdU was added to a final concentration of 10 μM. Then, EdU incorporation was detected after 24 h in accordance with the manufacturer's instructions.

3C Library Construction

Cells (10^7) were resuspended and fixed in 2% formaldehyde in culture medium for 10 min at room temperature, then quenched by

(B) Efficient knockdown of *Mid1* with two different shRNAs. ESCs were transfected with plasmids expressing shMid1_1 and shMid1_2, as well as a control vector. After a 5-day puromycin selection, cells were harvested for qRT-PCR. Three biological replicates per sample were assayed. Data are shown as the mean ± SEM (n = 3). ***p < 0.001, t test.
(C) Proliferation assays for control and *Mid1* knockdown ESCs. After transfection and puromycin selection as described in (B), the surviving cells were used for proliferation assays. The y axis is cell number. Three biological replicates per sample were assayed. Data are shown as the mean ± SEM (n = 3). ***p < 0.001, t test.
(D) Western blots demonstrate Flag-tagged *Mid1* overexpression in WT, homozygous ΔPG and ΔG cells.
(E) Colony-forming assays of control and *Mid1* overexpression ESCs. Representative pictures are shown. mESCs (WT, two homozygous ΔG clones, two ΔPG ESCs clones) were transfected with overexpression vector. Transfected cells were selected and picked as single clone. C1 and C2 were two individual cell lines picked from ESCs that were transfected with control vector. M1 and M2 were two individual cell lines picked from ESCs that were transfected with the vector containing Mid1 coding sequence. Scale bars, 50 μm.
(F) The relative diameters of colonies (E) were measured and shown with a box-and-whisker plot. Three biological replicates per sample were assayed. Contrast in one-way ANOVA analysis was carried out. Data are shown as the mean ± SEM. *p < 0.05, ***p < 0.001.
(G) The top panel is the schematic illustration of the *Mid1* locus. Arrows (A–F) represent the PCR amplicons for 3C experiment. The bottom panel illustrates the 3C-qPCR results detecting interactions between the *5430416N02Rik* promoter and the *Mid1* locus in heterozygous and homozygous ΔG ESCs. Contrast in one-way ANOVA analysis was carried out. Data are shown as the mean ± SEM. **p < 0.01, ***p < 0.001.
(H) A working model for lncRNA *5430416N02Rik* to regulate the expression of *Mid1*. The lncRNA 5430416N02Rik gene locus collaborates with CAPs, such as CTCF, Cohesin, and Mediators, to stabilize the interactions between the 5430416N02Rik promoter and the *Mid1* locus, consequently activating *Mid1* expression.

0.125 M ice-cold glycine. Cells were centrifuged for 8 min at 320 × *g* at 4°C and washed once in 10 mL of cold PBS. All the supernatant was removed and the cell pellet was resuspended in 5 mL of cold Lysis Buffer (10 mM Tris, 10 mM NaCl, 5 mM $MgCl_2$, 0.1 mM EGTA, 1×protease inhibitors, 0.2% NP-40) and incubated for 10 min on ice. The cells were then centrifuged for 5 min at 600 × *g* at 4°C, and the supernatantwas removed. The cell pellet was resuspended once with 10 mL of cold PBS and centrifuged for 5 min at 600 × *g* at 4°C; the supernatant was discarded. The cell pellet was resuspended in 440 μL of Milli-Q, 60 μL of 10× RE buffer, and 7.5 μL of 20% SDS, and incubated for 1 h at 37°C while shaking at 900 rpm. Then, 50 μL of 20% Triton X-100 was added and incubated for 1 h at 37°C while shaking at 900 rpm. Restriction enzymes DpnII (NEB; 400 U) were added followed by incubation overnight at 37°C while shaking at 900 rpm. Another 200 U of DpnII was added and incubated for 6 h at 37°C while shaking at 900 rpm. Then, 40 μL of 20% SDS was added and incubated for 20 min at 65°C to inactivate the enzyme. The samples (~600 μL) were transferred to a 50-mL Falcon tube, 700 μL of 10× ligation buffer and 5.7 mL of Milli-Q were added to 7 mL. Triton X-100 (20%, 375 μL) was added followed by incubation for 1 h at 37°C while shaking gently. T4 ligase (100 U; Clontech) was added followed by incubation overnight at 4°C. Protease K (20 mg/mL, 15 μL, 300 μg) was added followed by de-crosslinking overnight at 65°C. RNase A (10 mg/mL, 30 μL, 300 μg) was added followed by incubation for 45 min at 37°C. DNA was then extracted with phenol-chloroform and the 3C library was quantified by NanoDrop.

Bacterial artificial chromosomes (BACs) (RP23-292L23, RP24-250A6, RP23-154O12, and RP24-385C17) were purchased from BACPAC and amplified with conventional protocols. Then, the pooling of BAC DNA (2.5 μg for each) was digested with DpnII. After ligation with T4 ligase, DNA was purified and quantified for qPCR. qPCR was performed with Bio-Rad iQ SYBR Green Supermix (Bio-Rad), with 50 ng templates of the 3C library and 10 ng of BAC control DNA. Then, the relative interaction frequency was calculated with the ΔΔCt method.

4C-Seq

The 3C library using HindIII (NEB) was constructed as above. Then, all the 3C library was digested in a 500 μL reaction with 50 U of DpnII and incubated overnight at 37°C. Then, 14 mL of the reaction mixture was ligated with 100 U of ligase at 16°C overnight followed by purification with phenol-chloroform. The samples were purified with the QIAquick PCR Purification Kit (QIAGEN) and quantified with NanoDrop. 4C templates (3.2 μg) were pre-mixed with 160 μL of 5× PCR buffer, 40 μL of dNTP (10 mM), 19.2 μL of forward primer (50 μM), 19.2 μL of reverse primer (50 μM), 11.2 μL of Expand Long Template Polymerase (Roche), and ddH_2O to 800 μL in total. The sample was separated into 16 reactions of 50 μL each, then PCR was with the following program: 2 min at 94°C, 10 sec at 94°C, 1 min at 55°C, 3 min at 68°C, 29 repeats, 5 min at 68°C, and 4°C forever. The samples were purified with a QIAquick PCR Purification Kit and run on agarose gel (2%) for 10 min. Gel excision (150–800 bp) was performed followed by extraction with a QIAquick Gel Extraction Kit (QIAGEN). The 4C library was then purified with a QIAquick PCR Purification Kit. The quality and concentration of the 4C library were checked with a bio-analyzer.

Two biological replicates were performed for each 4C-seq experiment. 4C libraries were then sequenced as 50 bp single-end reads using the HiSeq 2000 platform (BGI). 4C-seq data were analyzed using methods described elsewhere (Cai et al., 2016a, 2016b; van de Werken et al., 2012). To start with, sequencing reads with specific primer sequence were selected. Sub-fragments concatenated with primer sequences were mapped to the reference genome (NCBI37/mm9) with BWA. Mapped fragments were further aligned to a reduced "library" containing genome-wide HindIII sites. A "binarization" step was taken to minimize the PCR duplication bias. Then, a window-based model was used to identify significantly interacting regions. Each interacting site i was examined in the foreground window w with length lw (HindIII sites) and background window W with length LW (HindIII sites). The number of interacting sites for window (i,w) was denoted as Ci,w, and a *Z* score was assigned to the window based on the following formula:

$$zi = (Ci,w - \mu W)/\mathrm{sqrt}(\mu W(1 - \mu W/lw))$$

where μW is the expected number of interacting sites in window (i,w) calculated based on the total number of interactions in window (i,W).

A foreground window size of 500 and 100 was used in counting the number of ligated sites to define inter-chromosomal and intra-chromosomal interactions, respectively. To define intra-chromosomal interactions, we set the background window size to 3,000 to calculate the expected number of ligated sites in a foreground window. For inter-chromosomal interactions, the background window was set as the whole chromosome since no distance adjustment is needed. The calculated *Z* scores were compared with the standard Gaussian distribution and regions with p values smaller than 0.05 were taken as significant candidates.

Capture-C-Seq

The 3C library using DpnII was constructed as above. The 3C library was then sonicated to 100–300 bp and the library was constructed and indexed with the NEBNext DNA Library Prep Master Mix Set for Illumina (NEB). Then, 1 μg from each indexed library was pooled and then captured with two biotin-labeled probes using the SeqCap EZ Hybridization and Wash Kit (Roche). After amplification with P5 and P7 primers, the second capture step was performed with the same probes. The Capture-C library was then amplified with P5 and P7 primers followed by purification with the AMPure XP system (Beckman Coulter). The library size and the concentration were checked with the DNA bio-analyzer.

Two biological replicates were performed for each Capture-C experiment. The bar-coded Capture-C libraries were sequenced as 125 bp paired-end reads using the Illumina HiSeq 2500 platform. Capture-C data were analyzed with the pipeline proposed in Davies et al. Adaptor sequences were trimmed off from raw reads using Trim Galore (https://www.bioinformatics.babraham.ac.uk/projects/trim_galore/). Trimmed read pairs with one mate shorter than 20 bp or with a Phred quality score lower than 20 were discarded. Read pairs with central overlap were reconstructed into single reads using FLASH. Next, *in silico* restriction enzyme digestion was performed to identify junctions formed by distally interacting

sub-fragments and viewpoint sub-fragments. Sub-fragments were aligned to the reference genome (GRCh37/hg19) with bowtie2. Reads with the same composition of sub-fragments with regard to coordinate (chr:start-end) and strandness were considered PCR duplicates. Three classes of sub-fragments were defined. (1) "capture": sub-fragment mapped within a restriction fragment flanked by the designed probe pair (view point); (2) "proximity exclusion": sub-fragment mapped to a defined proximity exclusion region, which is 1 kb extended from the view point in two directions; and (3) "reporter": sub-fragment mapped outside the capture and proximity exclusion regions. The interaction frequency for each DpnII fragment was summarized and presented in a bedGraph file that could be visualized in IGV.

RIP-Seq

RIP was constructed with an EZ-Magna Nuclear RIP (Cross-Linked) Nuclear RNA-Binding Protein Immunoprecipitation Kit (Millipore), using antibodies of Klf4 (R&D, AF3158), CTCF (EMD Millipore, 07-729), Med12 (Bethyl Laboratories, A300-774A), Smc1a (Bethyl Laboratories, A300-055A) and Flag (Sigma, F1804). Libraries were then constructed with a NEBNext Ultra Directional RNA Library Prep Kit for Illumina (NEB). The library size and concentration were then checked with the DNA bio-analyzer.

Three biological replicates were performed for each RIP-seq experiment. The bar-coded RIP-seq libraries were sequenced as 100 bp paired-end reads using the Illumina HiSeq 4000 platform. Reads were mapped to the reference genome (NCBI37/mm9) using TopHat2 with a GTF file obtained from GENCODE supplied as gene model annotations (tophat2 –library-type fr-firststrand -G gencode.vM1.annotation.gtf). To adjust for PCR amplification bias from the library preparation, we estimated the complexity of the library and removed duplicates with the Picard (https://github.com/broadinstitute/picard) tool MarkDuplicates (REMOVE_DUPLICATES = true). HTSeq was used to quantitate the filtered reads mapped to each gene (htseq-count -f bam -r name -s reverse -t exon -i gene_name). We further normalized read counts by library size and fitted a generalized linear model with a negative binomial distribution using the DESeq2 R package. PCA was conducted with regularized log transformed data (Figure 1A). Lists of genes with differential transcripts abundance between RIP samples and input samples were obtained with the criteria of a Benjamini-Hochberg adjusted p value <0.05 and fold change (RIP versus input) >1.5. Differential genes were overlapped and shown in a Venn diagram (Figure 1B). We used the same kit to perform RIP-seq and RIPs for PCR in Figure S1G.

RNA-Seq

The RNA library was constructed with TruSeq Stranded Total RNA with Ribo-Zero Human/Mouse/Rat (Illumina). Three biological replicates were performed for each RNA-seq experiment. The bar-coded RNA-seq libraries were sequenced as 100 bp paired-end reads using the Illumina Hiseq 4000 platform. The analysis was performed similarly as in the RIP-seq analysis above. DESeq2 was used to perform normalization and regularized log transformation on read counts data. We applied Gene Cluster 3.0 (http://bonsai.hgc.jp/~mdehoon/software/cluster/software.htm) to cluster gene expression data. Genes that have at least one sample with an absolute value ≥ 1.0 and maximal difference of values between samples ≥ 1.0 were centered by mean and then subjected to hierarchical clustering. The similarity metric of correlation and the clustering method of average linkage were used. Clustering results were examined and visualized in Java Treeview (http://jtreeview.sourceforge.net/).

Knockdown, Knockin, Overexpression

Gene knockdown was performed with the pSUPER.puro system (Oligoengine) following the instructions of the manufacturer. In brief, for the expression of small hairpin RNA targeting the Mid1 gene, a 60-nt oligo containing the specific 19-nt targeting sequence was cloned into the BglII/XhoI site of the pSuper-puro vector. A control plasmid targeting GFP was constructed at the same time.

For the Flag knockin or ΔG and ΔPG cell lines, the targeting vector was cotransfected with Cas9 and two sgRNAs together with the donor DNA in order to achieve simultaneous deletion and replacement. ESC colonies were picked, expanded, and analyzed to identify deletions or insertions.

For gene overexpression, we used the PB system (SBI). Coding sequence (CDS) for the *Mid1* longest isoform was amplified from mouse cDNA then inserted into the PB Transposon vector using BglII and XhoI. For transgene overexpression, 5×10^5 mESCs were transfected with 2 μg of PB Transposon vector together with 1 μg of PB Transposase vector using Lipofectamine 3000 (Invitrogen) according to the manufacturer's instructions. Transfected cells were selected under 200 μg/mL hygromycin B (Invitrogen) for 7–8 days after transfection.

Proliferation Assay

Cells (1.5×10^5) were plated at day 0 in triplicate and passaged in a 1:10 ratio every 2 days. Cells were counted with a hemocytometer at each time point.

Colony-Forming Assay

A low density of cells (10^3 cells/cm^2) was plated and cultured for 5 days. Colonies were washed once with PBS solution and incubated with an Alkaline Phosphatase Substrate Kit III (Vector) for 20 min at room temperature. The size of colony was measured under a microscope.

qRT-PCR

Total RNA was extracted from cells using the RNeasy Mini Kit (QIAGEN). cDNA synthesis was performed using the iScript Reverse Transcription Supermix (Bio-Rad) following by qPCR using iQ SYBR Green Supermix (Bio-Rad) according to the manufacturer's instructions. Quantification of target genes was normalized with β-actin. At least three biological replicates per experiment were assayed. Three technical replicates were performed for each sample.

Western Blot

Cells were lysed in Lysis Buffer (Beyotime), and the protein concentration was measured using a BCA Protein Assay Kit (Beyotime) to ensure equal loading. The samples were resolved by SDS-PAGE and then transferred onto a polyvinylidene fluoride

membrane (Millipore). Membranes were probed with anti-Oct4 (Santa Cruz, sc-5279), anti-Sox2 (GeneTex, GTX101507), anti-Nanog (Bethyl Laboratories, A300-397A), anti-Flag (Sigma, F1804), and anti-β-tubulin (Huada, Beijing, AbM59005-37B-PU). Immunoreactivity was detected by ECL Plus (Beyotime). Digital images were taken with by the automatic chemiluminescence imaging analysis system.

DNA FISH

Both experimental and control BAC clones (5430416N02Rik: RP24-385C17; Mid1: RP23-154O12, RP24-250A6; Col1A1: RP23-75B6, RP23-321D21) were purified using a QIAGEN MidiPrep Kit (QIAGEN, Germantown, MD) and were further validated using locus-specific primer pairs. For probe generation, all the BAC DNAs were labeled with biotin-11-dUTP or digoxigenin-11-dUTP for 90 min at 15°C using the nick translation system (Enzo Life Sciences, Farmingdale, NY), and quenched with salmon sperm DNA and mouse Cot-1 DNA. Probes were then precipitated with ethanol and resuspended in deionized formamide and 2× hybridization buffer (2× saline-sodium citrate [SSC], 10% low molecular weight dextran sulfate, 0.1% Tween 20). Following a previously described protocol, E14 cells were fixed with 4% formaldehyde and permeabilized briefly with 0.5% Triton X-100 on ice. All the samples were denatured in 70% formamide/2× SSC (pH 7.2) for 30 min at 80°C and dehydrated sequentially with ice-cold ethanol. Slides were then hybridized with the probe pool in a humid chamber at 37°C overnight. After incubation, slides were washed in 50% formamide/2× SSC at 42°C and incubated with anti-DIG-rhodamine for 40 min at 37°C. After washing with 0.1% Tween 20/4× SSC, slides were then incubated with avidin-fluorescein (Vector Labs, Burlingame, CA) for 40 min at 37°C. Slides were then washed again in 0.1% Tween 20/2× SSC and counter-stained with DAPI before mounting. Images were collected using confocal microscopy (Zeiss, Jena, Germany). Overlapping fluorescein and rhodamine signals were considered co-localized loci.

Purification DNA by RNA Antisense Purification

RAP was performed as previously described with some minor modifications (Engreitz et al., 2013). In brief, ESCs were crosslinked on plates with 2 mM disuccinimidyl glutarate (Pierce) and then further crosslinked with 3% formaldehyde. Batches of 5 million cells were lysed on ice in 5 mL of Lysis Buffer 1 (10 mM HEPES [pH 7.5], 20 mM KCl, 1.5 mM $MgCl_2$, 0.5 mM EDTA, 1 mM Tris(2-carboxyethyl) phosphine (TCEP), 0.5 mM PMSF) for 15 min, then centrifuged at 3,300 × *g* for 5 min. Cell pellets were resuspended in 1 mL of Lysis Buffer 1 plus 0.1% NP-40 and dounced 20 times. After another spin, nuclei were lysed on ice in 600 μL of Lysis Buffer 2 (20 mM Tris [pH 7.5], 50 mM KCl, 1.5 mM $MgCl_2$, 2 mM TCEP, 0.5 mM PMSF, 100 U of Murine RNase Inhibitor, 0.4% sodium deoxycholate, 1% NP-40, 0.1% N-lauroylsarcosine) for 10 min. To solubilize chromatin, samples were sonicated with a 30-s ON, 30-s OFF procedure for 10 cycles at 4°C (Diagenode SA Picoruptor). To obtain DNA fragments of approximately 100–300 bp, samples were treated with 2.5 mM $MnCl_2$, 0.5 mM $CaCl_2$, and 100 U TURBO DNase (Ambion) at 37°C for 1 h. DNase digestion was stopped by addition of 10 mM EDTA and 5 mM EGTA on ice for at least 5 min. The lysate were diluted to hybridization conditions by adding 2× RAP hybridization buffer (1×: 20 mM Tris [pH 7.5], 7 mM EDTA, 3 mM EGTA, 150 mM LiCl, 1% NP-40, 0.2% N-laroylsarcosine, 0.1% sodium deoxycholate, 3 M guanidine thiocyanate, 2.5 mM TCEP). The lysate were centrifuged at 14,000 × *g* for 10 min. For the purification, denatured probes (ACA TGA TGC CAG AGC GTT AAC AAA AGG AGG ACC GAA TGG AAG CGA GAA CGT GCC CAC GG, CTC CCC ATG GCC ATG CTC GCC CAA AGC GCG ATG GCG CCG GAT TGC GCG GGA GCT GGC GT, TCC CGG CGC GCG CCA CCA TCG CCA GGG TCA GGC AGG CGT CAG CTC GGT TTC CAC CTC TC, GAG AGC TCC TGT GCC CCC TCG CCA TCC GTG ACC CAT TTG GCG CCA TTC TCC AAT CAG TC, GGA CAG CGG AGC AGT CCC ACT CAA GCT GCT GTG CAT CAC ATC CAC AGC GCC AGG TCA CA, AGA ACA CAA TTC CTT CTT TCA TTA GCC ACC TCC CAT CTT CAG AAA AAG CGT TCT CTC AT, ATT CCT TTC CTG TAT TTC GTA ATC ATC TTC CAA CGT CAC GCA TCT CTT CCA GGC ATC AC, ATC TTC AAA TTA ATG AAT ATT TCT GTA AGT GTT ACT CTT ACA AAA AAC ATC CTT TGA TT, AGG TCT CTT CTT CCA CAC TGT GGG TCC CAG GAA TTA ACG GAG GTC ACC AGG CCT TAT AT, TTT TTA ATT ACA TTT ATT TAC ATA TTG TGT ATA TGT GGT CAG AAG ACA ACT TTC AGG AG) were mixed with the heated lysate and incubated at 39°C for 4 h to capture target RNAs, then pre-cleared by adding 100 μL of streptavidin-coated C1 beads (Invitrogen) and incubating at 37°C for 1 h. We transferred the samples to a magnetic rack and washed them six times with RAP Wash Buffer (20 mM Tris [pH 7.5], 10 mM EDTA, 1% NP-40, 0.2% N-laroylsarcosine, 0.1% sodium deoxycholate, 3 M guanidine thiocyanate, 2.5 mM TCEP) at 45°C for 5 min. At this point, we used two different elution methods to examine the associated RNA or DNA. (1) Eluting for RNA qPCR: we eluted twice in 50 μL of RAP Elution Buffer (20 mM Tris [pH 7.5], 10 mM EDTA, 2% N-laroylsarcosine, 2.5 mM TCEP) by heating to 94°C for 5 min. We pooled the resulting eluates and reversed crosslinks by incubating at 65°C for 1 h after addition of 250 mM NaCl and 1 mg/mL Proteinase K (New England Biolabs). We purified RNA by isopropanol precipitation onto SILANE beads (Invitrogen) and treated with TURBO DNase (Ambion) before proceeding to qRT-PCR. (2) We eluted captured chromatin complexes and reversed crosslinks by adding 80 μL of RAP Elution Buffer plus 250 mM NaCl and 1 mg/mL Proteinase K to the oligo-bead complexes and incubated overnight at 65°C. DNA was purified with 1.8× volume AMPure XP beads and analyzed by qRT-PCR.

Primers

Detailed information on the primers used in the study is given in Table S3.

Statistical Analysis

Unless specified in the text, data were analyzed by unpaired two-tailed Student's t test. Statistically significant p values are indicated in the figures as follows ***$p < 0.001$, **$p < 0.01$, *$p < 0.05$.

Additional experimental procedures are provided in Supplemental Information.

ACCESSION NUMBERS

The accession number for the 4C-seq, Capture-C-seq, RIP-seq, and RNA-seq data reported in this paper is GEO: GSE133435.

SUPPLEMENTAL INFORMATION

Supplemental Information can be found online at https://doi.org/10.1016/j.stemcr.2020.02.002.

AUTHOR CONTRIBUTIONS

L.C. and W.L. contributed to the data interpretation and manuscript writing. T.Z. conceived and designed the project and performed most experiments. M.C. contributed to the bioinformatics. M.L. contributed to the cell-cycle analysis, the flow cytometry analysis of cell apoptosis and the EdU incorporation analysis. G.S. contributed to the purification DNA by RNA antisense purification. D.A. contributed to the Mid1 overexpression cell lines construction. B.M., G.L. and Y.S. contributed to the DNA FISH.

ACKNOWLEDGMENTS

W.L. was supported by the National Natural Science Foundation of China (grant no. 31530027) and the National Key R&D Program of China (grant no. 2017YFA0102600).L.C. was supported by the National Key R&D Program of China (grant no. 2018YFC1313003 and 2018YFA0107002), the National Natural Science Foundation of China (grant no. 31622038, 31671497 and 31871485), the 111 Project grant (B08011).

Received: July 6, 2019
Revised: February 9, 2020
Accepted: February 10, 2020
Published: March 10, 2020

REFERENCES

Apostolou, E., Ferrari, F., Walsh, R.M., Bar-Nur, O., Stadtfeld, M., Cheloufi, S., Stuart, H.T., Polo, J.M., Ohsumi, T.K., Borowsky, M.L., et al. (2013). Genome-wide chromatin interactions of the nanog locus in pluripotency, differentiation, and reprogramming. Cell Stem Cell *12*, 699–712.

Bergmann, J.H., Li, J., Eckersley-Maslin, M.A., Rigo, F., Freier, S.M., and Spector, D.L. (2015). Regulation of the ESC transcriptome by nuclear long noncoding RNAs. Genome Res. *25*, 1336–1346.

Cai, M., Gao, F., Lu, W., and Wang, K. (2016a). w4CSeq: software and web application to analyze 4C-seq data. Bioinformatics *32*, 3333–3335.

Cai, M., Kim, S., Wang, K., Farnham, P.J., Coetzee, G.A., and Lu, W. (2016b). 4C-seq revealed long-range interactions of a functional enhancer at the 8q24 prostate cancer risk locus. Sci. Rep. *6*, 22462.

Dixon, J.R., Jung, I., Selvaraj, S., Shen, Y., Antosiewicz-Bourget, J.E., Lee, A.Y., Ye, Z., Kim, A., Rajagopal, N., Xie, W., et al. (2015). Chromatin architecture reorganization during stem cell differentiation. Nature *518*, 331–336.

Dixon, J.R., Selvaraj, S., Yue, F., Kim, A., Li, Y., Shen, Y., Hu, M., Liu, J.S., and Ren, B. (2012). Topological domains in mammalian genomes identified by analysis of chromatin interactions. Nature *485*, 376–380.

Engreitz, J.M., Pandya-Jones, A., McDonel, P., Shishkin, A., Sirokman, K., Surka, C., Kadri, S., Xing, J., Goren, A., Lander, E.S., et al. (2013). The Xist lncRNA exploits three-dimensional genome architecture to spread across the X chromosome. Science *341*, 1237973.

Evans, M.J., and Kaufman, M.H. (1981). Establishment in culture of pluripotential cells from mouse embryos. Nature *292*, 154–156.

Guttman, M., Donaghey, J., Carey, B.W., Garber, M., Grenier, J.K., Munson, G., Young, G., Lucas, A.B., Ach, R., Bruhn, L., et al. (2011). lincRNAs act in the circuitry controlling pluripotency and differentiation. Nature *477*, 295–300.

Hacisuleyman, E., Goff, L.A., Trapnell, C., Williams, A., Henao-Mejia, J., Sun, L., McClanahan, P., Hendrickson, D.G., Sauvageau, M., Kelley, D.R., et al. (2014). Topological organization of multichromosomal regions by the long intergenic noncoding RNA Firre. Nat. Struct. Mol. Biol. *21*, 198–206.

Handoko, L., Xu, H., Li, G., Ngan, C.Y., Chew, E., Schnapp, M., Lee, C.W., Ye, C., Ping, J.L., Mulawadi, F., et al. (2011). CTCF-mediated functional chromatin interactome in pluripotent cells. Nat. Genet. *43*, 630–638.

Huang, J., Marco, E., Pinello, L., and Yuan, G.C. (2015). Predicting chromatin organization using histone marks. Genome Biol. *16*, 162.

Ji, X., Dadon, D.B., Powell, B.E., Fan, Z.P., Borges-Rivera, D., Shachar, S., Weintraub, A.S., Hnisz, D., Pegoraro, G., Lee, T.I., et al. (2016). 3D chromosome regulatory landscape of human pluripotent cells. Cell Stem Cell *18*, 262–275.

Kagey, M.H., Newman, J.J., Bilodeau, S., Zhan, Y., Orlando, D.A., van Berkum, N.L., Ebmeier, C.C., Goossens, J., Rahl, P.B., Levine, S.S., et al. (2010). Mediator and cohesin connect gene expression and chromatin architecture. Nature *467*, 430–435.

Kim, D.H., Marinov, G.K., Pepke, S., Singer, Z.S., He, P., Williams, B., Schroth, G.P., Elowitz, M.B., and Wold, B.J. (2015). Single-cell transcriptome analysis reveals dynamic changes in lncrna expression during reprogramming. Cell Stem Cell *16*, 88–101.

Ko, C.I., Fan, Y., de Gannes, M., Wang, Q., Xia, Y., and Puga, A. (2016). Repression of the aryl hydrocarbon receptor is required to maintain mitotic progression and prevent loss of pluripotency of embryonic stem cells. Stem Cells *34*, 2825–2839.

Kung, J.T., Kesner, B., An, J.Y., Ahn, J.Y., Cifuentes-Rojas, C., Colognori, D., Jeon, Y., Szanto, A., del Rosario, B.C., Pinter, S.F., et al. (2015). Locus-specific targeting to the X chromosome revealed by the RNA interactome of CTCF. Mol. Cell *57*, 361–375.

Li, Y.P., Duan, F.F., Zhao, Y.T., Gu, K.L., Liao, L.Q., Su, H.B., Hao, J., Zhang, K., Yang, N., and Wang, Y. (2019). A TRIM71 binding long noncoding RNA Trincr1 represses FGF/ERK signaling in embryonic stem cells. Nat. Commun. *10*, 1368.

Loewer, S., Cabili, M.N., Guttman, M., Loh, Y.H., Thomas, K., Park, I.H., Garber, M., Curran, M., Onder, T., Agarwal, S., et al. (2010). Large intergenic non-coding RNA-RoR modulates reprogramming of human induced pluripotent stem cells. Nat. Genet. *42*, 1113–1117.

Mondal, T., Rasmussen, M., Pandey, G.K., Isaksson, A., and Kanduri, C. (2010). Characterization of the RNA content of chromatin. Genome Res. *20*, 899–907.

Phillips-Cremins, J.E., Sauria, M.E., Sanyal, A., Gerasimova, T.I., Lajoie, B.R., Bell, J.S., Ong, C.T., Hookway, T.A., Guo, C., Sun, Y., et al. (2013). Architectural protein subclasses shape 3D organization of genomes during lineage commitment. Cell *153*, 1281–1295.

Quinn, J.J., and Chang, H.Y. (2016). Unique features of long non-coding RNA biogenesis and function. Nat. Rev. Genet. *17*, 47–62.

Tang, Z., Luo, O.J., Li, X., Zheng, M., Zhu, J.J., Szalaj, P., Trzaskoma, P., Magalska, A., Wlodarczyk, J., Ruszczycki, B., et al. (2015). CTCF-mediated human 3D genome architecture reveals chromatin topology for transcription. Cell *163*, 1611–1627.

Tu, J., Tian, G., Cheung, H.H., Wei, W., and Lee, T.L. (2018). Gas5 is an essential lncRNA regulator for self-renewal and pluripotency of mouse embryonic stem cells and induced pluripotent stem cells. Stem Cel. Res. Ther. *9*, 71.

van de Werken, H.J., de Vree, P.J., Splinter, E., Holwerda, S.J., Klous, P., de Wit, E., and de Laat, W. (2012). 4C technology: protocols and data analysis. Methods Enzymol. *513*, 89–112.

Wang, Y., Crawford, D.R., and Davies, K.J. (1996). adapt33, a novel oxidant-inducible RNA from hamster HA-1 cells. Arch. Biochem. Biophys. *332*, 255–260.

Wang, Y., Davies, K.J., Melendez, J.A., and Crawford, D.R. (2003). Characterization of adapt33, a stress-inducible riboregulator. Gene Expr. *11*, 85–94.

Wei, Z., Gao, F., Kim, S., Yang, H., Lyu, J., An, W., Wang, K., and Lu, W. (2013a). Klf4 organizes long-range chromosomal interactions with the oct4 locus in reprogramming and pluripotency. Cell Stem Cell *13*, 36–47.

Wei, Z., Huang, D., Gao, F., Chang, W.H., An, W., Coetzee, G.A., Wang, K., and Lu, W. (2013b). Biological implications and regulatory mechanisms of long-range chromosomal interactions. J. Biol. Chem. *288*, 22369–22377.

Yin, Y., Yan, P., Lu, J., Song, G., Zhu, Y., Li, Z., Zhao, Y., Shen, B., Huang, X., Zhu, H., et al. (2015). Opposing roles for the lncRNA haunt and its genomic locus in regulating HOXA gene activation during embryonic stem cell differentiation. Cell Stem Cell *16*, 504–516.

Zhang, H., Jiao, W., Sun, L., Fan, J., Chen, M., Wang, H., Xu, X., Shen, A., Li, T., Niu, B., et al. (2013). Intrachromosomal looping is required for activation of endogenous pluripotency genes during reprogramming. Cell Stem Cell *13*, 30–35.

Stem Cell Reports, Volume *14*

Supplemental Information

lncRNA *5430416N02Rik* Promotes the Proliferation of Mouse Embryonic Stem Cells by Activating *Mid1* Expression through 3D Chromatin Architecture

Tong Zhao, Mingyang Cai, Man Liu, Guangsong Su, Daniel An, Byoungsan Moon, Guochang Lyu, Yibo Si, Lingyi Chen, and Wange Lu

Supplemental Information

Supplemental Figures and Legends

Figure S1. Analysis of RIP-seq profiles. (Related to Figure 1)

Figure S2. The *5430416N02Rik* promoter is a chromatin interaction hub. (Related to Figure 2)

Figure S3. Both homozygous ΔPG and ΔG ESCs show reduced growth rates. (Related to Figure 3)

Figure S4. The transcriptional profile is perturbed more in homozygous ΔPG ESCs than in homozygous ΔG ESCs. (Related to Figure 3)

Figure S5. Both *5430416N02Rik* DNA and RNA interact with the *Mid1* locus. (Related to Figure 4)

Supplemental Tables

Table S1. Klf4-bound RNAs identified by RIP-seq; Lists of Klf4-bound lncRNAs, lncRNAs regulating ESC transcriptome, and lncRNAs activated during reprogramming. (Table S1.xlsx)

Table S2 *5430416N02Rik*-interacting sites and regions identified by 4C-seq; *5430416N02Rik*-interacting sites in heterozygous and homozygous ΔG ESCs identified by Capture-C seq; Differential *5430416N02Rik*-interacting sites between heterozygous and homozygous ΔG ESCs identified by Capture-C seq. (Table S2.xlsx)

Table S3. Differentially expressed genes in heterozygous and homozygous ΔPG and ΔG ESCs, as well as RA treated ESCs identified by RNA-seq; List of primers used in the study. (Table S3.xlsx)

Supplemental Figures and Legends

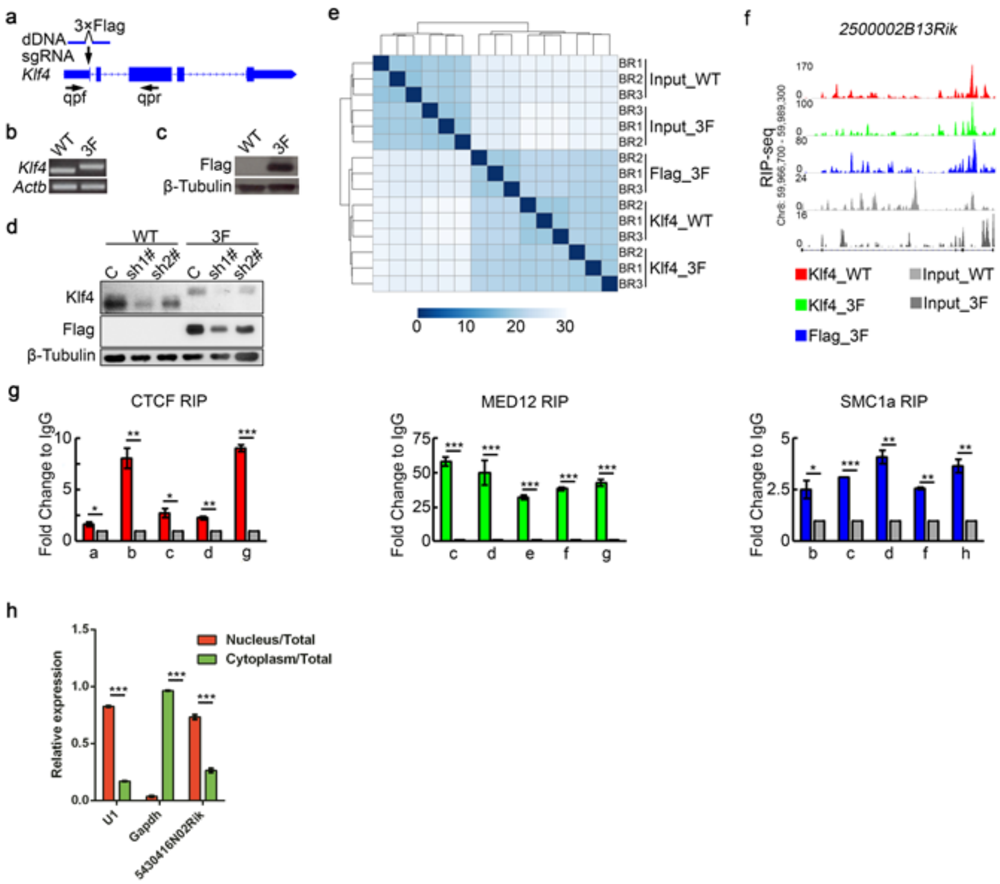

Figure S1. Analysis of RIP-seq profiles.

(a) Schematic illustration of the knock-in strategy at the *Klf4* locus. Blue rectangles represent *Klf4* exons. A donor DNA (dDNA) containing 3′ Flag sequence for *Klf4* N terminal knock-in is shown at the top. A vertical arrow indicates the sgRNA targeting site. Arrows marked with qpf and qpr are primers for *Klf4* RT-PCR.

(b) RT-PCR results demonstrate homozygous knock-in of 3×Flag at the *Klf4* locus. PCR products using qpf and qpr primers show a 66 bp shift in 3F cells, compared with WT cells, due to the 3×Flag knock-in.

(c) Western Blot analysis confirms Flag expression in 3F cells.

(d) Western Blot analysis confirms the correct integration at the *Klf4* locus. WT and 3F cells were transfected with plasmids expressing *Klf4* shRNAs (sh1# and sh2#) as

well as a control vector. Cells were harvested 48 hours after transfection and processed for Western Blot with Klf4, Flag, and β-Tubulin antibodies.

(e) Clustered heatmap using all genes shows the sample-to-sample similarity between RIP-seq profiles. Three biological replicates are shown as BR1, BR2 and BR3. Complete hierarchical clustering is performed, basing on the Euclidean distances between samples.

(f) Representative signals at the *2500002B13Rik* locus from Klf4_WT (red), Klf4_3F (green) and Flag_3F (blue) RIP-seq, as well as input RNAs (grey).

(g) RIP qRT-PCR verifies the binding between CTCF, MED12, SMC1a and *5430416N02Rik* RNA. RIP assays were performed with CTCF antibody, MED12 antibody, SMC1a antibody and IgG antibody in WT ESCs. Eight DNA amplicons (a-h) are illustrated as short black bars in Figure 1e. Data are shown as the mean ± SEM (n=3).*p<0.05, **p<0.01, ***p<0.001.*t*-test.

(h) Cytoplasmic/nuclear fractionation analysis of mESCs shows that 5430416N02Rik is mainly located in the nuclear. U1 and Gapdh serve as positive ctrl of nucleus and cytoplasm, respectively. Data are shown as the mean ± SEM (n=3). ***p<0.001, *t*-test.

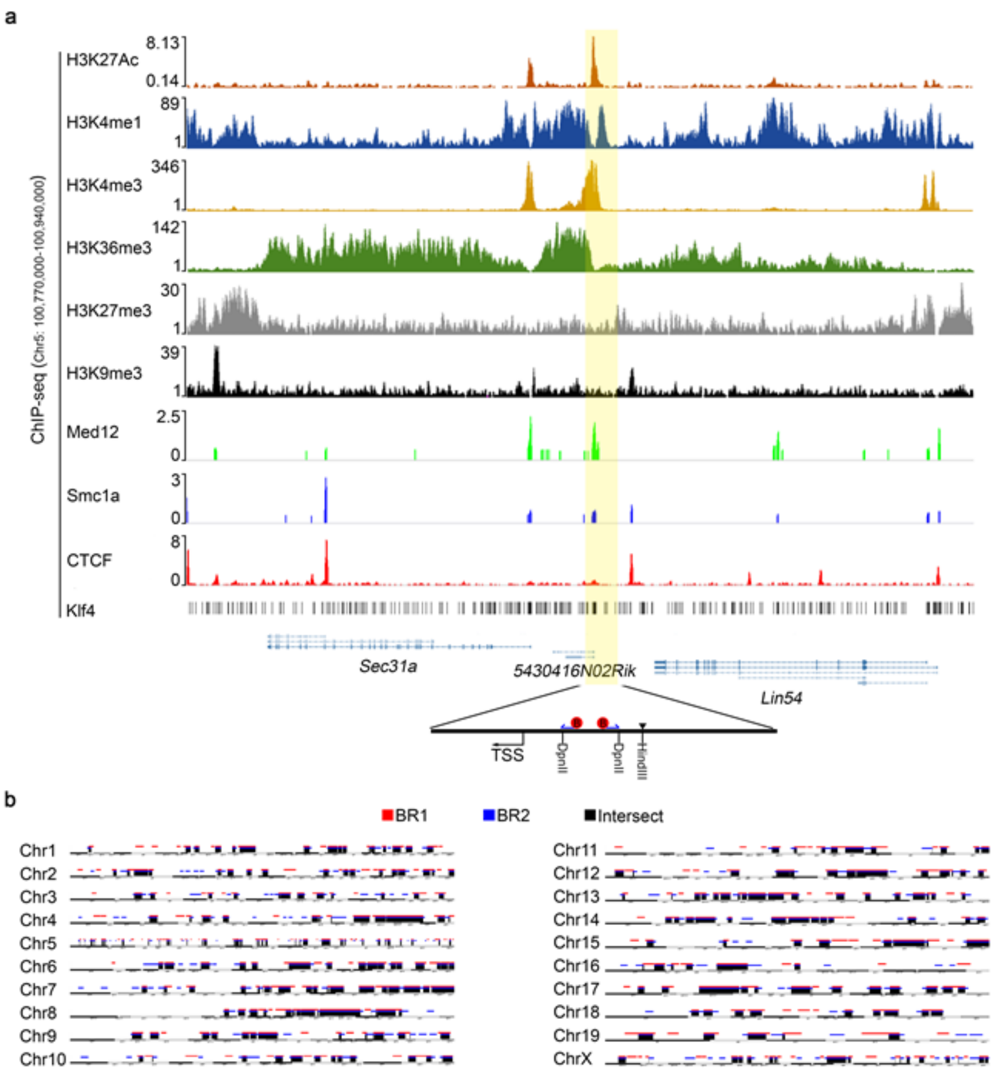

Figure S2. The *5430416N02Rik* promoter is a chromatin interaction hub.

(a) ChIP-seq enrichment patterns for histone modifications and chromatin architectural proteins at the *5430416N02Rik* locus. Tracks assembled in mm9 are displayed in the order of H3K27ac (ENCFF001KFN, ENCODE), H3K4me1 (ENCFF001ZGW, ENCODE), H3K4me3 (ENCFF001ZPH, ENCODE), H3K36me3 (ENCFF001ZHS, ENCODE), H3K27me3 (ENCFF001ZJJ, ENCODE), H3K9me3 (ENCFF001ZHH, ENCODE), Med12 (Pool of GSM560345 and GSM560346, GEO), Smc1a (Pool ofGSM560341 and GSM560342, GEO), CTCF (GSM918748, GEO) and Klf4 (GSM288354, GEO). The *5430416N02Rik* promoter region is zoomed into

and displayed by a bold black line at the bottom. Transcription orientation of *5430416N02Rik* is indicated by a black arrow. Blue arrows with red cycle at DpnII sites represent two biotin-labeled probes for Capture-C. A black triangle at the HindIII site indicates the bait for 4C-seq.

(b) Line plots show regions interacting with the *5430416N02Rik* promoter in each chromosome. Interacting regions identified in the two biological replicates of 4C-seq are shown with red and blue lines, respectively. Black boxes mark overlapped interacting regions from two biological replicates.

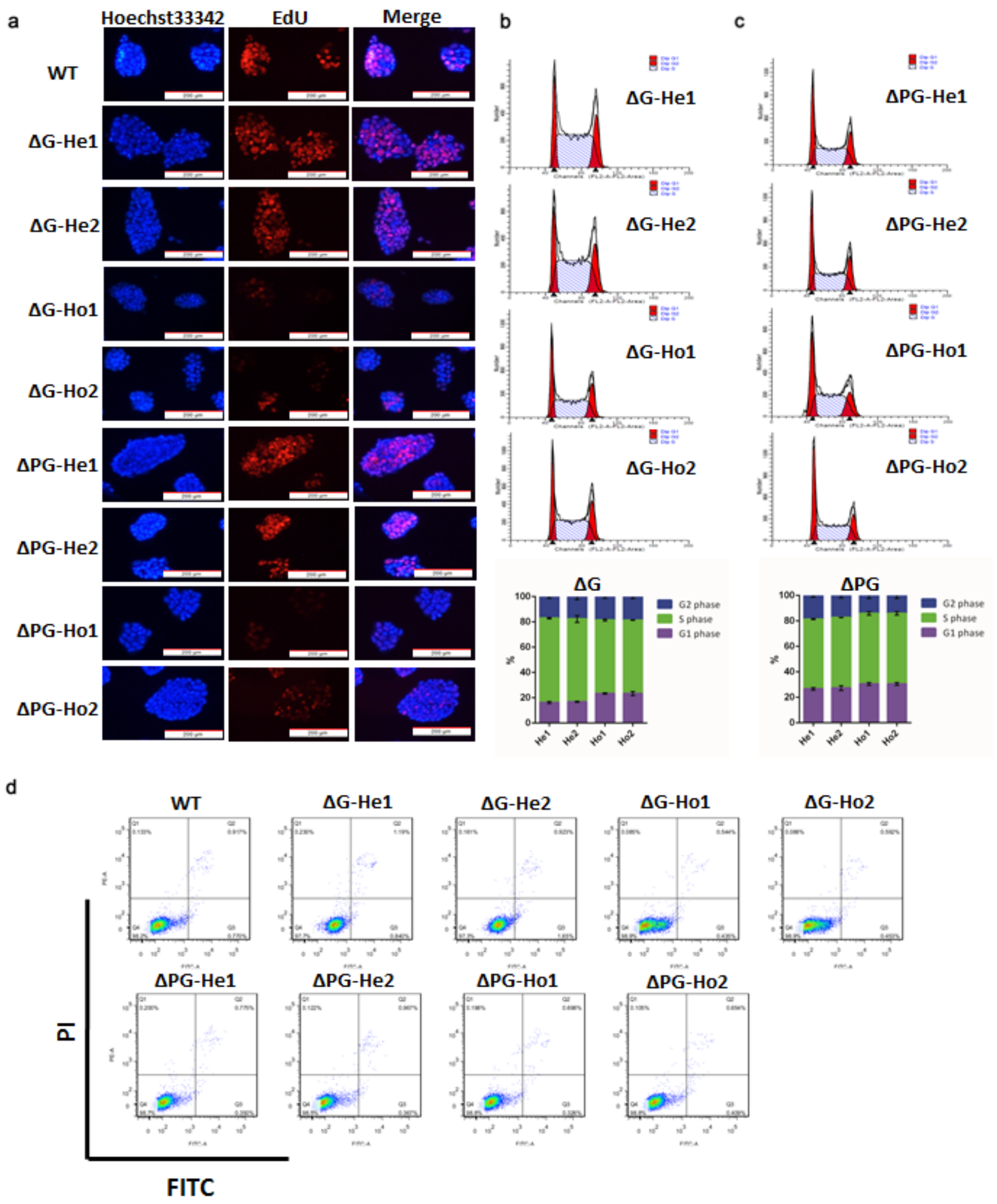

Figure S3. Both homozygous ΔPG and ΔG ESCs show reduced growth rates.

(a) Images of EdU incorporation analysis in WT, ΔG and ΔPG ESCs. Scale bar: 200 µm.

(b-c) Cell cycle analysis and quantification of cell cycle phases in ΔG (b) and ΔPG (c) ESCs.

(d) Cell apoptosis analysis.

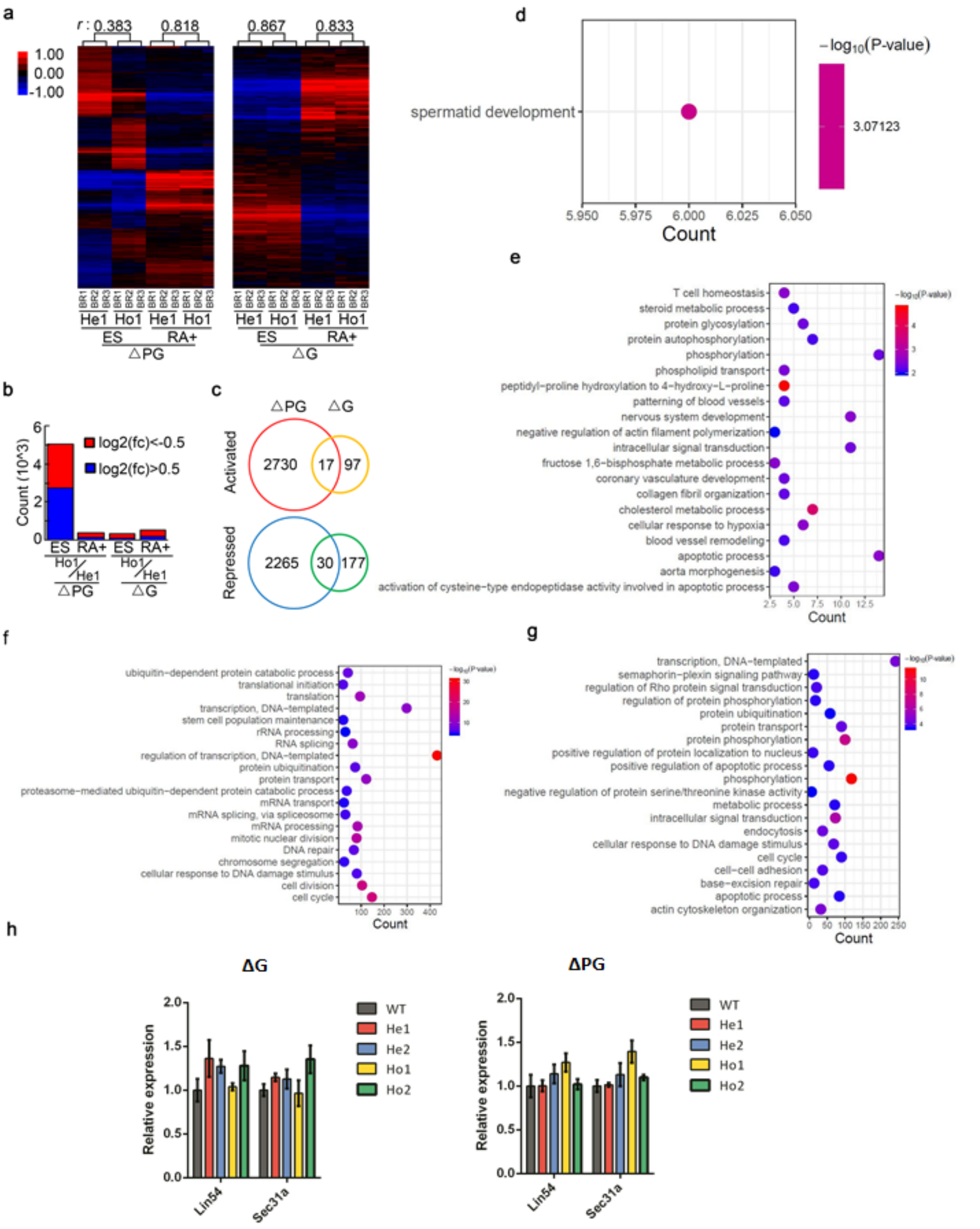

Figure S4. The transcriptional profile is perturbed more in homozygous ΔPG ESCs than in homozygous ΔG ESCs.

(a) Clustering of differentially regulated genes in heterozygous and homozygous ΔPG and ΔG ESCs, as well as RA treated ESCs. ESCs are treated with RA (2 μM) and

DMSO for 48 hours, and harvested for RNA-seq. Three biological replicates are included. Pearson correlation coefficient (*r*) is calculated and shown at the top.

(b) Numbers of activated or repressed genes in homozygous ΔPG and ΔG cells, in comparison to their heterozygous counterparts.

(c) Venn diagrams of activated (top) and repressed genes (bottom) in homozygous ΔPG and ΔG ESCs, in comparison to their heterozygous counterparts.

(d-g) GO analysis of biological progress of genes differentially up-regulated in homozygous ΔG cells (d), down-regulated in homozygous ΔG cells (e), up-regulated in homozygous ΔPG cells (f), down-regulated in homozygous ΔPG cells (g).

(h) Relative mRNA levels of two neighbor genes of 5430416N02Rik in homozygous ΔG and ΔPG and the control cells.

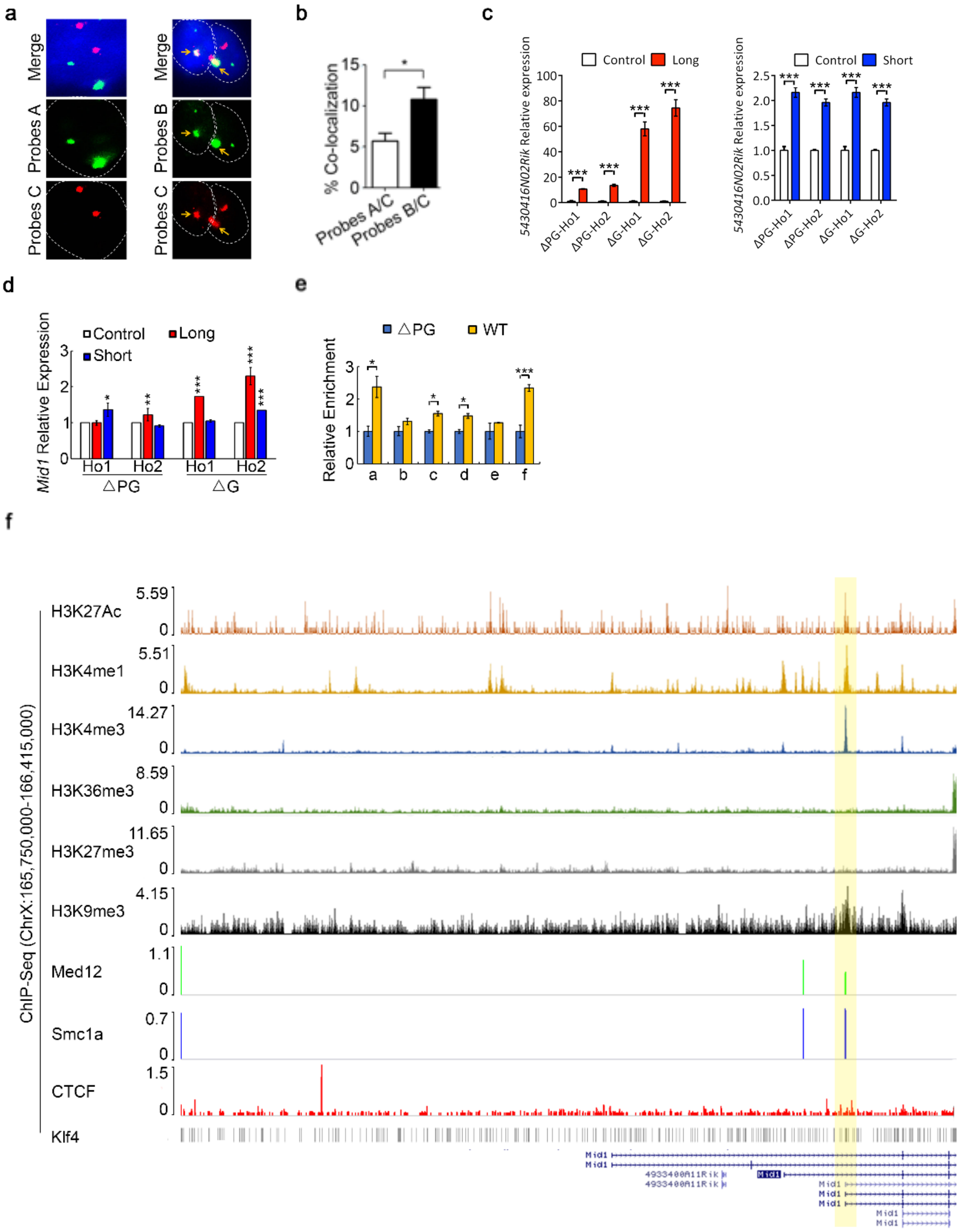

Figure S5. Both *5430416N02Rik* DNA and RNA interact with the *Mid1* locus.

(a) Representative images of DNA FISH show co-localizations of 5430416N02Rik with Col1A1 (left panel) or Mid1 (right panel). Probes A: Col1A1, green, BAC clones (RP23-75B6 and RP23-321D21). Probes B: Mid1, green, BAC clones (RP23-154O12 and RP24-250A6). Probes C: 5430416N02Rik, red, BAC clone (RP24-385C17).

(b) Quantification of the co-localization of 5430416N02Rik with Col1A1 or Mid1

(n=4, more than 50 nuclei were counted for each experiment). Data are shown as the mean ± SEM. *p<0.05,

(c) Relative mRNA levels of two isoform of 5430416N02Rik in homozygous ΔG and ΔPG ESCs that overexpressed two isoform of 5430416N02Rik. Data are shown as the mean ± SEM (n=3). ***p<0.001.*t*-test.

(d) Relative mRNA levels of Mid1 in homozygous ΔG and ΔPG ESCs that overexpressed two isoform of 5430416N02Rik. Data are shown as the mean ± SEM (n=3).*p<0.05, **p<0.01, ***p<0.001.*t*-test.

(e) Following RAP assay, interactions between *5430416N02Rik* RNA and DNA regions of *Mid1* were detected by qRT-PCR. DNA amplicons (a-f) are illustrated as in Figure 4g. Data are shown as the mean ± SEM. *p<0.05, ***p<0.001.*t*-test.

(f) ChIP-seq enrichment patterns for histone modifications and chromatin architectural proteins at the *Mid1* locus. Tracks assembled in mm9 are displayed in the same way of Figure S2a.
